# Fault-tolerant attitude control of the satellite in the presence of simultaneous actuator and sensor faults

**DOI:** 10.1038/s41598-023-48243-w

**Published:** 2023-11-27

**Authors:** Ali Reza Fazlyab, Farhad Fani Saberi, Mansour Kabganian

**Affiliations:** 1 Department of Mechanical Engineering, Amirkabir University of Technology, Tehran, 15875-4413 Iran; 2 Aerospace Science and Technology Institute, Amirkabir University of Technology, Tehran, 15875-4413 Iran

**Keywords:** Aerospace engineering, Mechanical engineering

## Abstract

In this paper, a robust attitude control algorithm is developed based on backstepping sliding mode control for a satellite using reaction wheels and thrusters that can perform its mission despite faulty actuators. In this method, the actuator dynamics have been considered to design the controller and the asymptotic stability of the proposed algorithm has been proven based on the Lyapunov theory. The designed controller can converge the attitude of the system into the desired path in the presence of faulty actuators. Then a fault-tolerant attitude estimation system is designed based on federated unscented Kalman filters that can be effectively employed to detect and isolate sensor faults. Finally, the performance of the designed attitude estimation and controller is investigated by simulation in the presence of both actuator and sensor faults.

## Introduction

Modern systems depend upon automatic control systems to satisfy their performance and safety requirements. A traditional feedback control design might not perform satisfactorily for a complex system, or it should become unstable even when there’s a malfunction within the operation of actuators, sensors, or other system components. To overcome such weaknesses, new methods were developed to resist fault occurrence in component performance and to take care of optimal performance and stability. This can be essential for systems that are sensitive to safety, like aircraft, spacecraft, atomic energy plants, and unsafe chemical processing plants. In such systems, the results of a minor fault within the system components are often catastrophic. Therefore, providing high reliability, safety, fault tolerance, and optimal performance is mostly necessary. These sorts of control systems are commonly called fault-tolerant control systems, abbreviated as FTCS. More specifically, FTCSs are control systems that can adapt to the presence of a fault or component failure. They’re ready to maintain the overall system stability and supply acceptable performance in the event of such faults.

A spacecraft needs several subsystems to accomplish its mission. One of the foremost important of those subsystems is the attitude determination and control system. The spacecraft attitude determination and control system can measure and determine the attitude and maintain the system within the desired direction by using different combinations of actuators and sensors. Because attitude determination and control (ADCS) is one of the foremost vital subsystems of a spacecraft, a fault in this system can have serious consequences.

The fault detection and diagnosis (FDD) mechanisms used in the attitude determination and control subsystems are only limited to model-based mechanisms and data-based mechanisms.

By reviewing the references, the model-based method is more used than the data-based method in the system of determining and controlling the attitude of the spacecraft, which is due to the special dynamic characteristics of the spacecraft, which are mentioned below:

1. The possibility of relatively accurate modeling of the dynamics of the spacecraft
2. A relatively complete understanding of the input–output information of spacecraft and their interactions with the environment
3. Compatibility with fault-compensating control techniques
4. The effectiveness of the method in all operating conditions of the spacecraft

To further emphasize this issue, the following expression from reference^1^ is used.

The model-based fault detection and isolation system has been used mostly in mechanical, electrical, and aerospace systems, while the data-based fault detection and isolation techniques have been used more in applications such as chemical systems due to the complexity and lack of access to accurate models.

Although model-based mechanisms have more advantages than other methods, their use for both attitude determination and control of spacecraft leads to the following problems.

When the actuators have a fault, the attitude determination system does not work properly and they suffer a severe drop in the accuracy of the attitude determination and when the sensors have a fault, the attitude control system will not work properly. Also, the simultaneous fault in the actuators and sensors cannot be compensated.

To avoid this problem, turning to passive fault-tolerant control methods including robust control has been significant.

The passive methods employed in the attitude control subsystem are robust methods that are utilized in the conditions before the fault occurs and after it occurs, and thus won’t require the FDD mechanism or rearrangement.

The simplicity of calculation and robustness to disturbances and faults have made the sliding mode control very interesting. It should be emphasized that in reality satellites are affected by various external disturbances and sliding mode control as a robust control has shown its ability to deal with external disturbances well^2^.

In^3^ Various kinds of sliding mode control and its application to fault tolerant control are proposed. The adaptive control method could be a solution that’s presented within the^4^ for a satellite and uses the standard structure consisting of 4 reaction wheels as an actuator. Reference^5^ offers another passive fault compensation strategy supported by adaptive sliding mode control. In this reference, the satellite is considered a flexible body, in which the momentum of inertia is comparatively estimated and utilized in the control law. Within the reference^6^ a sliding mode control law is employed to control the attitude of a satellite. This control law is meant to be proof against the consequences of faults in satellite actuators in addition to external disturbances. Have used robust and neural network controllers as fault-tolerant control^7,8^.

In^9^ sliding mode control is provided along with the fuzzy method in the presence of external disturbances and actuator input dead-zone. In^10–12^ an adaptive nonsingular terminal sliding mode control is designed to solve the attitude control of flexible spacecraft.

During the last decades, various works have been done for fault tolerant control of satellites, including neural network control^13^, adaptive control^14–16^, robust control^8,17^ etc. The recent development of spacecraft attitude fault tolerant control systems has been explained in^18^.

Although all the authors of the mentioned works have guaranteed the performance of their system, none of them have considered the limitations and dynamics of the actuators and the simultaneity of faults in the sensor and actuator. Therefore, in this article, considering the dynamics of the actuator in the design of the control and comparing the effect of making the dynamics more accurate is one of the main goals of the design.

In this paper, to take into account the effect of the dynamics of the actuators, the backstepping control is used, which can coordinate with any control, and its asymptotic stability along with a sliding mode control has been proven based on the Lyapunov theory.

Another important part of the satellite attitude control system is its sensors, and sensor faults can cause irreparable damage. Therefore, different algorithms are used to estimate the satellite attitude using multiple sensors. Algorithms that are commonly used to estimate the attitude of satellites are, Kalman filters^19^, extended Kalman filters^20,21^, unscented Kalman filters^22^, and particle filters^23^

The extended Kalman filter has been widely used to estimate spacecraft attitude. However, the linearization of the satellite nonlinear system with the help of the Taylor series causes poor estimation^24^, hence UKF is known as an estimator that produces better results than EKF. Instead of linearizing the equations, UKF generates a limited number of points called sigma points. These points are changed to a series of new points using nonlinear equations and provide a more accurate estimate. For a system that has multiple sensors, there are two different schemes for processing sensor data using filters. Centralized Kalman filter, and Decentralized Kalman filter^25^. In the centralized method, the data of all the sensors is processed in a central unit, and it causes problems when the filter is saturated with data. In the decentralized method, local filters estimate the data of each sensor and produce the final optimal data with the help of a fusion criterion. Determining and isolating the fault is also easier due to the parallel structure. Also, by increasing the speed and accuracy of the fault-tolerant attitude estimation system, the system will work properly if there is a simultaneous fault in the actuators and sensors.

In this article, after expressing the dynamic and kinematic relations of the spacecraft, the arrangement and relations governing the dynamics of the actuators employed in the spacecraft, are discussed such as the thruster and reaction wheel. Since model-based systems, despite their many capabilities, aren’t able to simultaneously investigate faults in the actuator and sensor, first the passive and robust sliding mode method is employed to complete faults within the actuators, so its performance is improved by considering the actuator dynamics in its design. Then a fault-tolerant attitude estimation is designed by using federated unscented Kalman filters. Finally, the performance of the designed fault tolerant attitude estimation is examined by simulation in the presence of fault in star tracker and gyro sensors.

## Dynamics and kinematics of the spacecraft

For mathematical modeling, the spacecraft is taken into account as a rigid body. the final equation of rotational motion of a rigid body in space (Euler equation) within the presence of reaction wheels is as follows^26^.

1 $$ \underline{{\dot{\omega }}} = J^{ - 1} \left[ {\underline{J\omega } \times } \right]\,\underline{\omega } + J^{ - 1} \underline{u} $$

2 $$ \left[ {\underline{J\omega } \times } \right] = \left[ {\begin{array}{*{20}c} 0 & { - \left( {J\omega } \right)_{3} } & {\left( {J\omega } \right)_{2} } \\ {\left( {J\omega } \right)_{3} } & 0 & { - \left( {J\omega } \right)_{1} } \\ { - \left( {J\omega } \right)_{2} } & {\left( {J\omega } \right)_{1} } & 0 \\ \end{array} } \right] $$

In relation (1), $\underline{\omega } \in R^{3}$ is the angular velocity of the spacecraft relative to the body coordinate system and $\underline{u}$ is the control torque. $J$ is the moment of inertia of the spacecraft without the moment of inertia of the reaction wheels, which will be explained in more detail in future sections.

To express the kinematic equations of the spacecraft, Eq. (3) is used^27^ which $\left( {\,\underline{{q_{v} }} ,q_{4} } \right) \in R^{3} \times R$ are quaternions and represent the orientation of the spacecraft, and the relation (4) is always correct.

3 $$ \underline{{\dot{q}_{v} }} = \frac{1}{2}\left( {\,q_{4} I_{3 \times 3} + \left[ {\underline{{q_{v} }} \times } \right]\,} \right)\,\underline{\omega } ,\quad \dot{q}_{4} = - \frac{1}{2}\underline{{q_{v} }}^{T} \underline{\omega } $$

4 $$ \underline{{q_{v} }}^{T} q_{v} + q_{4}^{2} = 1 $$

In Eq. (4), $\underline{{q_{v} }} : = \left[ {q_{1} ,\,\,q_{2} ,\,\,q_{3} } \right]^{T} \in R^{3}$ is the vector part of the quaternions and $q_{4} \in R$ is the scalar part. Also, $\left[ {\underline{a} \times } \right]$ is a vector operator, as defined below

5 $$ \left[ {\underline{a} \times } \right] = \left[ {\begin{array}{*{20}c} 0 & { - a_{3} } & {a_{2} } \\ {a_{3} } & 0 & { - a_{1} } \\ { - a_{2} } & {a_{1} } & 0 \\ \end{array} } \right] $$

### Attitude control actuators

Actuators are tools for applying control torque to the spacecraft. The spacecraft attitude control system can use various actuators like momentum wheels, reaction wheels, moment control gyros, thrusters, and magnetic coils. These actuators will be used alone or together, looking at the sort of stabilization, control targets, mass, size, power consumption, and therefore the ability to come up with torque and angular velocity within the spacecraft.

In the following, the reaction wheel and the thruster are introduced and their dynamic equations are going to be extracted.

#### Reaction wheel

**Figure 1:**
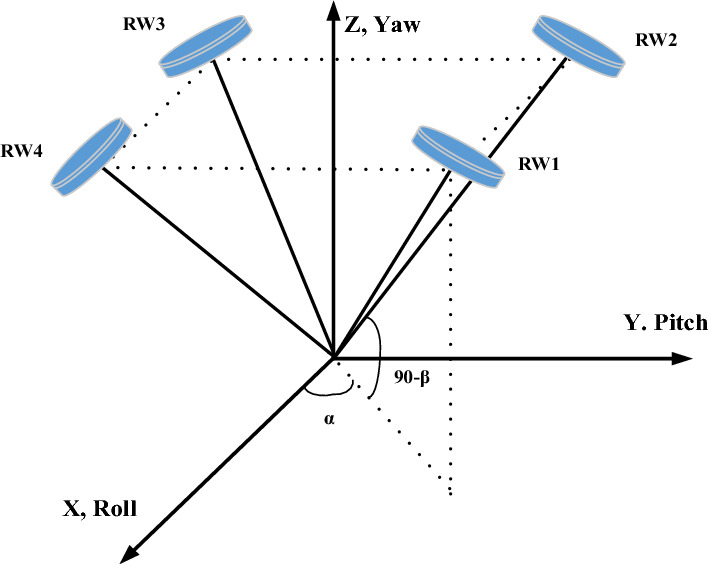
Reaction wheel structure.

The reaction wheel is an electrical motor that rotates the disk in the wrong way to the spacecraft. The wheel makes up a small proportion of the spacecraft's mass and provides precise control to the spacecraft. Reaction wheels are used for smooth and high-precision maneuvers. How the reaction wheel is arranged within the spacecraft plays a really important role in creating torque to control the spacecraft. Different arrangements are presented supported by three or four reaction wheels. The three reaction wheels with rotating axes parallel to the axes of the spacecraft body provide a simple arrangement for the reaction wheels. Because of the separation of the three-axis dynamics of the spacecraft, the design for each of the axes is often done independently, but if one of the wheels is broken, then it's impracticable to properly control all axes of the spacecraft. For this reason, a fourth wheel is sometimes installed to extend the reliability of the whole system. the extra wheel is installed in such a way that its axis isn’t in line with any of the three main axes and might create control torque around all three axes of the body. in this paper, four reaction wheels with a pyramidal structure (Fig. 1) are considered spacecraft actuators in such a way that all four wheels have the flexibility to form control torque around all axes.

The position of the axis of the wheels is expressed by a matrix. Therefore, the torque from the wheels is calculated as Eq. (6).

6 $$ \dot{h}_{w} = \left[ {\begin{array}{*{20}c} {\dot{h}_{wx} } & {\dot{h}_{wy} } & {\dot{h}_{wz} } \\ \end{array} } \right] = C\dot{h}_{a} $$

In Eq. (6), the matrix indicates the position of the wheels and $\dot{h}_{a}$ are the torques resulting from the rotation of the reaction wheels.

In this structure, the matrix ***c*** will be calculated from Eq. (7)

7 $$ C = \left[ {\begin{array}{*{20}c} {\cos \alpha \sin \beta } & { - \sin \alpha \sin \beta } & { - \cos \alpha \sin \beta } & {\sin \alpha \sin \beta } \\ {\sin \alpha \sin \beta } & {\cos \alpha \sin \beta } & { - \sin \alpha \sin \beta } & { - \cos \alpha \sin \beta } \\ {\cos \beta } & {\cos \beta } & {\cos \beta } & {\cos \beta } \\ \end{array} } \right] $$

In relation (7) $C_{i}$ ($i = 1,2,3,4$) are an axial vector of each wheel. It is also intended $\alpha = 45^{ \circ }$ and $\beta = 54.57^{ \circ }$ to optimize energy consumption in the wheels^28^.

##### Dynamic model of the reaction wheel

The structure of the reaction wheel consists of a flywheel, a fixed axis, and a DC motor. When the control signal enters the reaction wheel, the DC motor creates the greatest limitation. The spacecraft dynamics are controlled by the control torque applied to the spacecraft by the reaction wheels. To produce this torque, the voltage of the wheels must be controlled. This is done by designing the attitude controller taking into account the dynamics of the actuator motor. The differential equation of the circuit armature of a DC motor is given in Eq. (8)^29^.

8 $$ L_{a} \dot{I}_{a} + R_{a} I_{a} + K_{b} \Omega = v $$

where $R_{a}$,$L_{a}$,$K_{b}$,$\Omega$,$I_{a}$ and $v$ represents the armature resistance, armature inductance, reverse EMF constant, motor angular velocity, armature current, and applied voltage, respectively. On the other hand, the torque generated by the motor is directly proportional to the armature current.

9 $$ u = K_{m} I_{a} $$

In Eq. (9), $K_{m}$ is the torque constant of the motor. By placing Eq. (9) in (8), a linear general equation for the reaction wheel dynamics model is obtained, which is shown in Eq. (10).

10 $$ \frac{{L_{a} }}{{K_{m} }}\dot{u} + \frac{{R_{a} }}{{K_{m} }}u + K_{b} \Omega = v\quad \Rightarrow \quad T\underline{{\dot{u}}} + A\underline{u} + d = \underline{v} $$

In relation (10), $\underline{u} \in \Re^{M}$ is the actual input control vector, $T = T^{T} > 0$ is a diagonal matrix with a positive time constant, $d$ is finite Input disturbances and $A$ is a positive constant value. Disturbances enter the spacecraft directly as external disturbances ($T_{EXT}$) and affect the accuracy of the spacecraft’s attitude control and stability, so the spacecraft dynamics equation becomes the relation (11) in the presence of the reaction wheel model.

11 $$ \left\{ {\begin{array}{l} {\underline{{\dot{\omega }}} = J^{ - 1} \left( {\left[ {\underline{J\omega } \times } \right]\underline{\,\omega } } \right) + J^{ - 1} \underline{u} + J^{ - 1} T_{EXT} } \\ {T\underline{{\dot{u}}} + A\underline{u} + d = \underline{v} } \\ \end{array} } \right. $$

#### Thrusters

These actuators are engines that run on liquid or solid fuels and generate power by generating propulsion for control. On–off thrusters are commonly used for fast attitude maneuvers. Thrusters are capable of generating a continuing pulse torque, so controlling the optimum attitude with the thruster actuator could be a challenge because this pulsed torque causes a steady-state error within the system. The main methods for controlling the Reaction thruster are bang-bang control and Pulse modulation. Implementing the Bang Bang control method is straightforward but increases the thruster’s fuel consumption. Because fuel consumption could be a determining thing about the lifetime of a spacecraft, bang bang control isn’t an honest thanks to controlling thrusters. Therefore, the employment of Pulse modulation is more important because of the reduction in fuel consumption. The pulse modulation sends a sequence of pulses to the thrusters by adjusting the amplitude and frequency of the pulse. Pulse modulation includes a Pseudo-rate modulator^30^, an integral-pulse frequency modulator^31,32^, and pulse width-pulse frequency^33–36^. A pulse width-pulse frequency thanks to advantages like near-linear performance, high accuracy, and therefore the ability to regulate the bandwidth and frequency of the pulse is more useful.

**Figure 2:**
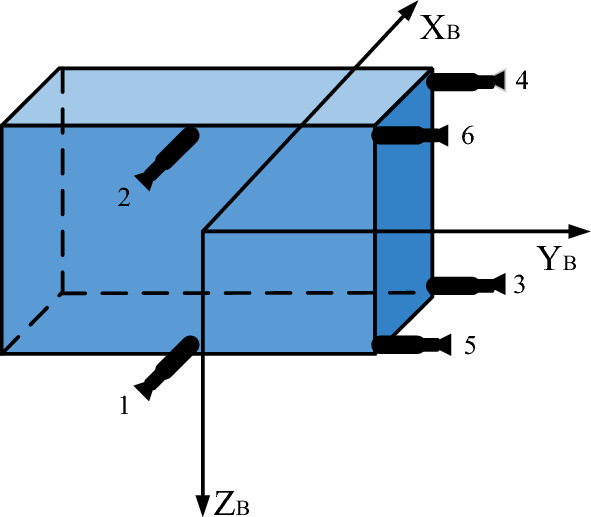
Six-thruster structure for spacecraft.

Naturally, the algorithm for transferring commands of control torques to the torque of every thruster depends on the arrangement of the thrusters. The algorithm presented in this paper relies on the structure shown in Fig. 2.

**Figure 3:**
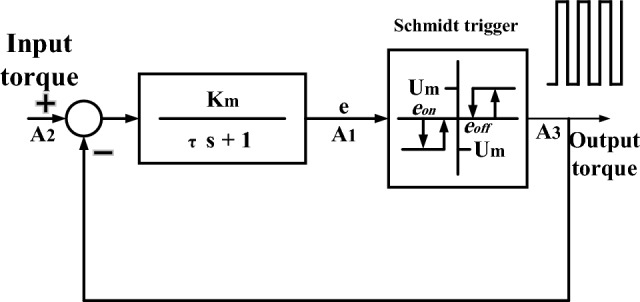
Schmidt trigger block.

In this paper, a pulse width-pulse frequency is used to convert continuous control commands to an on/off signal, which is suitable for a reaction thruster. Optimal values of The pulse width-pulse frequency parameters are given in^37^. Figure 3 shows the pulse width-pulse frequency block diagram.

**Figure 4:**
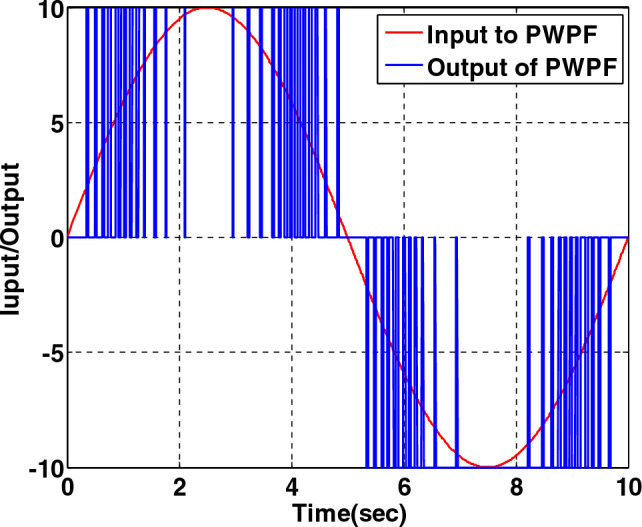
Converting a continuous sine wave to pulsed torque by PWPF.

In the Schmidt trigger block, when the input is positive and greater than $U_{on}$, the output is equal to $U_{m}$ which is the maximum output torque of the thruster. While for positive input and less than $U_{m}$ the output is zero. The same is true for negative values with the inverse sign. Figure 4 shows how to convert continuous torque to pulse by the pulse width-pulse frequency as an example.

##### Dynamic modeling of thruster

**Figure 5:**
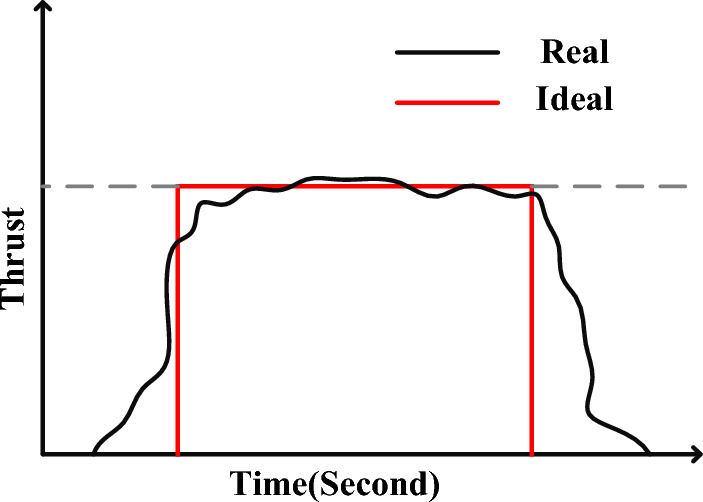
The real thrust profile compared to the square pulse.

In the simple thruster model, the thrust profile is assumed to be a square pulse, but in practice, the output torque of the thruster is quite different, as shown in Fig. 5.

**Figure 6:**
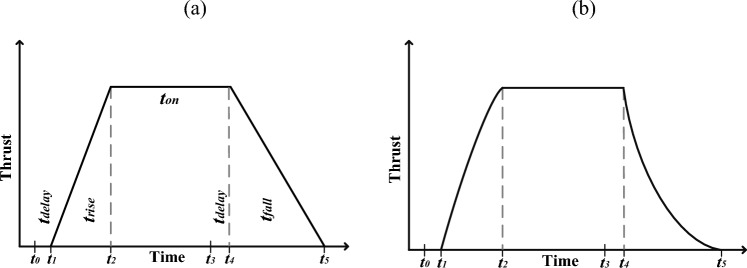
Trapezoidal approximation of the thrust profile.

The Goddard Space Flight Research Center typically uses a trapezoidal approximation shown in Fig. 6a^38^. In Fig. 6, the delay time ($t_{delay} = t_{1} - t_{0} \approx t_{4} - t_{3}$) is due to mechanical and electrical factors (such as the time of sending electrical signals and the delay in opening and closing the door of the thrust valves). The time from reaching ten percent of the thrust to ninety percent is called the rise time ($t_{rise}$) and the time from reaching ninety percent of the thrust to ten percent is called the settling time ($t_{fall}$). $t_{on}$ indicates the time when the production thrust is above ninety percent of the power of the thrust. Another model of the profile is created by substituting exponential functions at the edges of Fig. 6a, as seen in Fig. 6b. Although it is not possible to produce the profile in Fig. 5 accurately, the closer the approximations are to the actual model, the more accurately the controller can be designed.

**Figure 7:**
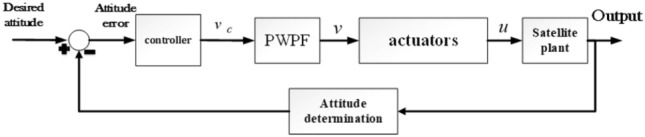
How the actuator model is placed in the closed-loop structure of the spacecraft control attitude.

In^39^ a linear model for the thruster is presented by Eq. (12) which $\underline{u} \in \Re^{M}$ is the actual vector of control input, $T = T^{T} > 0$ is a diagonal matrix with positive time constants. A demonstration of this model is given in Fig. 7.

12 $$ T\underline{{\dot{u}}} + \underline{u} = \underline{v} $$

As can be seen, by considering $A = d = 1$ in relation (10) we reach relation (12). Therefore, Eq. (10) can be considered as a general relation for the dynamics of both the reaction wheel and the thruster actuators. For this purpose, in the next section, a back-stepping sliding mode controller will be designed using Eq. (10).

## Design of fault-tolerant condition controller for a spacecraft

In this section, the three-axis attitude control is designed for the spacecraft in two stages.

Step 1: A sliding mode controller without considering the actuator dynamics.
Step 2: A back-stepping sliding mode controller considering the actuator dynamics.

Then, the simulation results of these two fault-tolerant controllers using thrusters and reaction wheel alone are presented.

### Design of sliding mode controller

In this section, a sliding mode controller is designed without considering the dynamic model of the actuator. For this purpose, the error dynamics are defined as follows.

If $$q_{e} = \left[ {\begin{array}{*{20}c} {\underline{{q_{ve} }} } & {q_{4e} } \\ \end{array} } \right]^{\,T}$$ indicates the error of the optimal attitude of the spacecraft relative to the body coordinate system, then the error is obtained from Eq. (13).

13 $$ q_{e} = q \otimes q_{d}^{ - 1} $$

In relation (13), $q_{d}^{ - 1}$ is the inverse of the desired quaternion and $\otimes$ is the operator of the quaternion multiplication. For both groups of quaternions, the error dynamics is defined as (14)^40^.

14 $$ \left[ {\begin{array}{*{20}c} {\underline{{\dot{q}_{ve} }} } \\ {\dot{q}_{4e} } \\ \end{array} } \right] = \frac{1}{2}\left[ {\begin{array}{*{20}c} {q_{4e} I_{3 \times 3} + \left[ {\,\underline{{q_{ve} }} \times } \right]} \\ { - \underline{{q_{ve} }}^{T} } \\ \end{array} \,} \right]\;\underline{{\omega_{e} \left( t \right)}} ,\;\;\underline{{\omega_{e} }} = \underline{\omega } - \underline{{\omega_{d} }} $$

where, $\underline{{\omega_{d} }}$ indicates the desired velocity of the body angles, which is considered zero, and therefore we will have:

15 $$ \underline{{\omega_{d} }} = \underline{0} \to \underline{{\omega_{e} }} = \underline{\omega } $$

Therefore, the derivative of the desired angular velocity will be obtained from the following equation.

16 $$ \underline{{\dot{\omega }_{e} }} = \underline{{\dot{\omega }}} = - J^{ - 1} \left[ {\underline{\omega } \times } \right]\,\,J\underline{\omega } + J^{ - 1} \underline{u} + J^{ - 1} T_{EXT} $$

Now, we will show that the sliding mode control law presented in Eq. (17) can stabilize the spacecraft system (Eqs. 1 and 3).

17 $$ \underline{u} = \left[ {\,\underline{\omega } \times } \right]\,\hat{J}\underline{\omega } - \frac{1}{2}\hat{J}C\,\left( {\,q_{4e} I_{3 \times 3} + \left[ {\underline{{q_{ve} }} \times } \right]\;} \right){\kern 1pt} {\kern 1pt} \underline{\omega } - \beta \left( {\underline{\omega } \,,\,q_{e} } \right)\,\underline{sat} \,\left( {{\raise0.7ex\hbox{${\underline{s} }$} \!\mathord{\left/ {\vphantom {{\underline{s} } \varepsilon }}\right.\kern-0pt} \!\lower0.7ex\hbox{$\varepsilon $}}} \right) $$

In relation (17), $$\beta {\kern 1pt} \left( {\,\underline{\omega } \,,\,q_{e} } \right)$$ is an upper limitation for system uncertainties and $$\underline{sat} {\kern 1pt} \left( {{\raise0.7ex\hbox{${\underline{s} }$} \!\mathord{\left/ {\vphantom {{\underline{s} } \varepsilon }}\right.\kern-0pt} \!\lower0.7ex\hbox{$\varepsilon $}}} \right)$$ is defined in relation (18).

18 $$ sat\left( {s_{i} \,,\,\varepsilon_{i} } \right) = \left\{ {\begin{array}{*{20}c} 1 & {for} & {s_{i} > \varepsilon_{i} } \\ {{\raise0.7ex\hbox{$s$} \!\mathord{\left/ {\vphantom {s \varepsilon }}\right.\kern-0pt} \!\lower0.7ex\hbox{$\varepsilon $}}} & {for} & {\left| {s_{i} } \right| < \varepsilon_{i} } \\ { - 1} & {for} & {s_{i} < - \varepsilon_{i} } \\ \end{array} } \right. $$

To prove the stability of the controller, first, a linear sliding surface is defined according to Eq. (19) in which the vector elements S belong to the set of real numbers and C is a diagonal matrix with scalar and positive elements.

19 $$ \underline{s} = \underline{\omega } + C\,\underline{{q_{ve} }} $$

The sliding surface derivative is obtained using Eq. (14) as Eq. (20).

20 $$ \underline{{\dot{s}}} = \underline{{\dot{\omega }}} + C\,\underline{{\dot{q}_{ve} }} = - J^{ - 1} \left[ {\,\underline{\omega } \times } \right]\,{\kern 1pt} J\underline{\omega } + J^{ - 1} \underline{u} + J^{ - 1} T_{EXT} + \frac{1}{2}C\,\left( {\,q_{4e} I_{3 \times 3} + \left[ {\underline{{q_{ve} }} \times } \right]\,} \right){\kern 1pt} {\kern 1pt} {\kern 1pt} \underline{\omega } $$

To reduce the phenomenon of chatting and energy consumption, the control input is divided into two parts, switching and continuous.

21 $$ \underline{u} = \underline{{u_{con} }} + \underline{{u_{swi} }} $$

Now, we consider $\underline{{u_{con} }}$ according to Eq. (22).

22 $$ \underline{{u_{con} }} = \left[ {{\kern 1pt} \underline{\omega } \times } \right]\,\hat{J}\underline{\omega } - \frac{1}{2}\hat{J}C\,\left( {\,q_{4e} I_{3 \times 3} + \left[ {\,\underline{{q_{ve} }} \times } \right]\,} \right){\kern 1pt} {\kern 1pt} \underline{\omega } $$

To find the switching part, we put Eq. (22) in Eq. (20) and we will have it.

23 $$ \underline{{\dot{s}}} = - J^{ - 1} \left[ {\underline{\omega } \times } \right]J\underline{\omega } + J^{ - 1} \left[ {\underline{\omega } \times } \right]\hat{J}\underline{\omega } + \frac{1}{2}C{\kern 1pt} \,\left( {\,q_{4e} I_{3 \times 3} + \left[ {\underline{{q_{ve} }} \times } \right]{\kern 1pt} } \right){\kern 1pt} \underline{\omega } - \frac{1}{2}J^{ - 1} \hat{J}\,C\,\left( {\,q_{4e} I_{3 \times 3} + \left[ {\underline{{q_{ve} }} \times } \right]\,} \right){\kern 1pt} {\kern 1pt} \,\underline{\omega } + J^{ - 1} T_{EXT} + J^{ - 1} \underline{{u_{swi} }} $$

We assume that the inertia matrix is in the form of $J = \hat{J} + \Delta {\kern 1pt} J$ in which $\hat{J}$ a fixed matrix and $\Delta {\kern 1pt} J$ indicates its uncertainty. Therefore, relation (23) is turned into relation (24):

24 $$ \underline{\delta } = - J^{ - 1} \left[ {\underline{\omega } \times } \right]\Delta {\kern 1pt} J\,\underline{\omega } + \frac{1}{2}J^{ - 1} \Delta {\kern 1pt} J\,C\,\left( {\,q_{4e} I_{3 \times 3} + \left[ {\underline{{q_{ve} }} \times } \right]\,} \right){\kern 1pt} {\kern 1pt} \underline{\omega } + J^{ - 1} T_{EXT} \;\;\underline{{\dot{s}}} \mathop = \limits^{def} \underline{\delta } + J^{ - 1} \underline{{u_{swi} }} $$

To determine the switching term, the upper limit of $J\underline{\delta }$ is assumed to be equal to $\alpha \left( {\underline{\omega } \,,\,q_{e} } \right)$ and therefore the switching term is obtained according to Eq. (25).

25 $$ \beta \,\left( {\underline{\omega } \,,\,q_{e} } \right) \ge \alpha \,\left( {\underline{\omega } \,,\,q_{e} } \right) + \beta_{0} \;\;\underline{{u_{swi} }} = - \beta \left( {\underline{\omega } \,,\,q_{e} } \right)\,sign\,\left( {\underline{s} } \right) $$

Another trick to reduce the chatting phenomenon is to use the saturation function instead of the sign function (according to Eq. (26)).

26 $$ sat\left( {s_{i} \,,\,\varepsilon_{i} } \right) = \left\{ {\begin{array}{*{20}c} 1 & {for} & {s_{i} > \varepsilon_{i} } \\ {{\raise0.7ex\hbox{$s$} \!\mathord{\left/ {\vphantom {s \varepsilon }}\right.\kern-0pt} \!\lower0.7ex\hbox{$\varepsilon $}}} & {for} & {\left| {s_{i} } \right| < \varepsilon_{i} } \\ { - 1} & {for} & {s_{i} < - \varepsilon_{i} } \\ \end{array} } \right. $$

$$ \underline{u} = \left[ {\underline{\omega } \times } \right]\hat{J}\underline{\omega } - \frac{1}{2}\hat{J}C\,\left( {\,q_{4e} I_{3 \times 3} + \left[ {\underline{{q_{ve} }} \times } \right]\,} \right){\kern 1pt} {\kern 1pt} \underline{\omega } - \beta {\kern 1pt} \left( {\underline{\omega } \,,\,q_{e} } \right)\,sat{\kern 1pt} \left( {{\raise0.7ex\hbox{${\underline{s} }$} \!\mathord{\left/ {\vphantom {{\underline{s} } \varepsilon }}\right.\kern-0pt} \!\lower0.7ex\hbox{$\varepsilon $}}} \right) $$

Now, to prove the asymptotic stability of the system, Lyapunov’s candidate function is considered as $V_{1} \left( s \right) = \frac{1}{2}\underline{s}^{T} \underline{s}$ The derivative of the Lyapunov function is calculated as follows

27 $$ \begin{gathered} \dot{V}_{1} \left( {\underline{s} } \right) = \underline{s}^{T} \underline{{\dot{s}}} = \underline{s}^{T} \left[ {\,\underline{\delta } + J^{ - 1} \,\underline{{u_{swi} }} } \right] \le \left\| {\underline{s} } \right\|\;\left\| {\underline{\delta } } \right\| + \underline{s}^{T} J^{ - 1} \underline{{u_{swi} }} \hfill \\ \le \left\| {\underline{s} } \right\|\;\left\| {\underline{\delta } } \right\| - \underline{s}^{T} J^{ - 1} \beta {\kern 1pt} \left( {\underline{\omega } \,,\,q_{e} } \right)\,sign\,\,\left( {\underline{s} } \right) \le \left\| {\underline{s} } \right\|\left\{ {\,\left\| {\underline{\delta } } \right\| - J^{ - 1} \beta {\kern 1pt} \,\left( {\underline{\omega } \,,\,q_{e} } \right)} \right\} \hfill \\ \le \left\| {\underline{s} } \right\|\;\left\| {J^{ - 1} } \right\|\left\{ {\,\left\| J \right\|\,\left\| {\underline{\delta } } \right\| - \beta \,\left( {\underline{\omega } \,,\,q_{e} } \right)\,} \right\} \le - \left\| {\underline{s} } \right\|\;\left\| {J^{ - 1} } \right\|\;\left\| {\beta_{0} } \right\|\, = - W\left( {\underline{s} } \right) \hfill \\ \end{gathered} $$

In relation (27), $W\left( {\underline{s} } \right)$ is a positive definite function. Therefore $\dot{V}_{1} \left( s \right)$ will be the negative definite and the asymptotic stability of the system will be proved. In the next section, the controller will be improved by adding a dynamic model of the actuator to the designed controller system.

### Design of back-stepping attitude controller—sliding mode

In this section, the actuator dynamic model presented in Eq. (10) is considered to design the attitude controller. To design the back-stepping sliding mode controller, the controller designed in the previous section is modified and then it is shown that the control law presented in relation (28) will stabilize. The system equations (dynamic equation in relation (1) and actuator dynamic in relation (10))

28 $$ \underline{v} = T\left[ { - k_{1} \underline{z} + \underline{{\dot{\phi }}} \,\left( {\underline{\omega } ,q_{e} } \right) + T^{ - 1} A\underline{u} + T^{ - 1} d} \right] $$

In Eq. (28), $T$ is a diagonal matrix with positive elements described in Actuator Dynamics (Eq. 10) $\underline{\phi } \left( {\underline{\omega } ,q_{e} } \right)$ is a feedback control that can stabilize system dynamics, and here the same sliding mode control of the previous section is considered. $\underline{u}$ is the control torque applied to the spacecraft. The relation between $\underline{u}$ and $\underline{v}$ is given in relation (10) and t $\underline{z} = \underline{u} - \underline{\phi }$.

First, by changing the variable $T\underline{{\dot{u}}} + A\underline{u} + d = w$ the dynamics equation of the system and the actuator becomes as relation (29)

29 $$ \left\{ {\begin{array}{l} {\underline{{\dot{\omega }}} = J^{ - 1} \left( { - \left[ {\underline{\omega } \times } \right]\,J\underline{\omega } + \underline{{T_{EXT} }} } \right) + J^{ - 1} \underline{u} } \\ {\underline{{\dot{u}}} = \underline{w} } \\ \end{array} } \right. $$

Equation (29) can also be written as Eq. (30)

30 $$ \left\{ {\begin{array}{l} {\underline{{\dot{\omega }}} = J^{ - 1} \left( { - \left[ {\underline{\omega } \times } \right]\,J\underline{\omega } + \underline{{T_{EXT} }} + \underline{\phi } \left( {\underline{\omega } } \right)} \right) + J^{ - 1} \left( {\underline{u} - \underline{\phi } \left( {\underline{\omega } } \right)} \right)} \\ {\underline{{\dot{u}}} = \underline{w} } \\ \end{array} } \right. $$

Note, that the stability of the first term to the right of Eq. (30) is guaranteed by the relation control law (31), which is the sliding mode controller of the previous section.

31 $$ \underline{\phi } \left( {\underline{\omega } ,q_{e} } \right) = \left[ {\underline{\omega } \times } \right]\hat{J}\underline{\omega } - \frac{1}{2}\hat{J}C\,\left( {\,q_{4e} I_{3 \times 3} + \left[ {\underline{{q_{ve} }} \times } \right]\,} \right){\kern 1pt} {\kern 1pt} \underline{\omega } - \beta \left( {\underline{\omega } \,,\,\underline{{q_{e} }} } \right)\,\,sat{\kern 1pt} {\kern 1pt} \left( {{\raise0.7ex\hbox{${\underline{s} }$} \!\mathord{\left/ {\vphantom {{\underline{s} } \varepsilon }}\right.\kern-0pt} \!\lower0.7ex\hbox{$\varepsilon $}}} \right) $$

With two changes of variables $\underline{z} = \underline{u} - \underline{\phi }$ and $\underline{p} = \underline{w} - \underline{{\dot{\phi }}}$ relation (30) becomes relation (32) and internal dynamics are asymptotically stable.

32 $$ \left\{ {\begin{array}{l} {\underline{{\dot{\omega }}} = J^{ - 1} \left( { - \left[ {\underline{\omega } \times } \right]J\underline{\omega } + T_{EXT} + \underline{\phi } \left( {\underline{\omega } } \right)} \right) + J^{ - 1} \underline{z} } \\ {\underline{{\dot{z}}} = \underline{w} - \underline{{\dot{\phi }}} \left( {\underline{\omega } } \right) = \underline{p} } \\ \end{array} } \right. $$

To prove the asymptotic stability of the whole system, the Lyapunov candidate function is defined as Eq. (33) by adding a certain positive definite term to the Lyapunov function of the sliding mode controller in the previous section, and its derivative will be obtained as Eq. (34).

33 $$ V_{a} = \frac{1}{2}\underline{s}^{T} \underline{s} + \frac{1}{2}\underline{z}^{T} \underline{z} $$

34 $$ \begin{array}{*{20}c} {\dot{V}_{a} = \underline {s}^{T} \underline{{\dot{s}}} + \underline {z}^{T} \underline{{\dot{z}}} } \\ { \Rightarrow \dot{V}_{a} = \underline {s}^{T} \left( {\underline {\delta } + J^{ - 1} \underline{{u_{swi} }} } \right) + \underline {z}^{T} \underline {p} } \\ { \Rightarrow \dot{V}_{a} \le - W\left( {\underline {s} } \right) + \underline {z}^{T} \underline {p} } \\ \end{array} $$

By selecting $\underline{p}$ as relation (35), $\dot{V}_{a}$ is determined as a negative definite and the asymptotic stability of the system is guaranteed.

35 $$ \begin{array}{*{20}c} {\underline {p} = - k_{1} \underline {z} } \\ { \Rightarrow \;\dot{V}_{a} \le - W\left( {\underline {s} } \right) - k_{1} \underline {z}^{T} \underline {z} } \\ { \Rightarrow \dot{V}_{a} < 0} \\ \end{array} $$

In relation (35), $k_{1}$ is a constant and positive value. Therefore, it was observed that the control input ($\underline{v}$) in relation (28) ensures the stability of the system in the presence of actuator dynamics. In the next section, the simulation results for both controllers in the presence of the dynamics of the thrusters and the reaction wheels are presented and compared with each other.

## Simulation of sliding mode control and back-stepping sliding mode control

In this section, to investigate the presence of faults in the actuators as well as the effect of considering the actuator dynamics in the controller design, simulation results for both thrusters and reaction wheels are presented. The design parameters for all simulations are given in Table 1.

**Table 1:** Systems parameter.

Quantity	Parameter	
$diag\,\left( {1000,500,700} \right)\,kg m^{2}$	$$\left[ {\begin{array}{*{20}c} {J_{x} } & {J_{y} } & {J_{z} } \\ \end{array} } \right]$$	Satellite dynamics
$$\left[ {\begin{array}{*{20}c} 0 & 0 & 5 \\ \end{array} } \right]\deg$$	$$\left[ {\begin{array}{*{20}c} {\phi_{0} } & {\theta_{0} } & {\psi_{0} } \\ \end{array} } \right]$$ initial conditions	
$$\left[ {\begin{array}{*{20}c} {20} & {25} & 0 \\ \end{array} } \right]\deg$$	$$\left[ {\begin{array}{*{20}c} \phi & \theta & \psi \\ \end{array} } \right]$$ desired attitude	
2	$K_{m}$	pwpf
0.5	$\tau$	
1	$U_{on}$	
0.1	$U_{off}$	
10	$U_{m}$	
0.75 (N m)	Maximum torque	Reaction wheel
470 (W)	Maximum power	
38.85(A)	Maximum current	
6000 (RPM)	Maximum angular velocity	
0.1	$\varepsilon$	Controller parameters
− 0.5	$\lambda$	
0.5	$k$	
15	$k_{1}$

(A) Reaction wheel.

**Figure 8:**
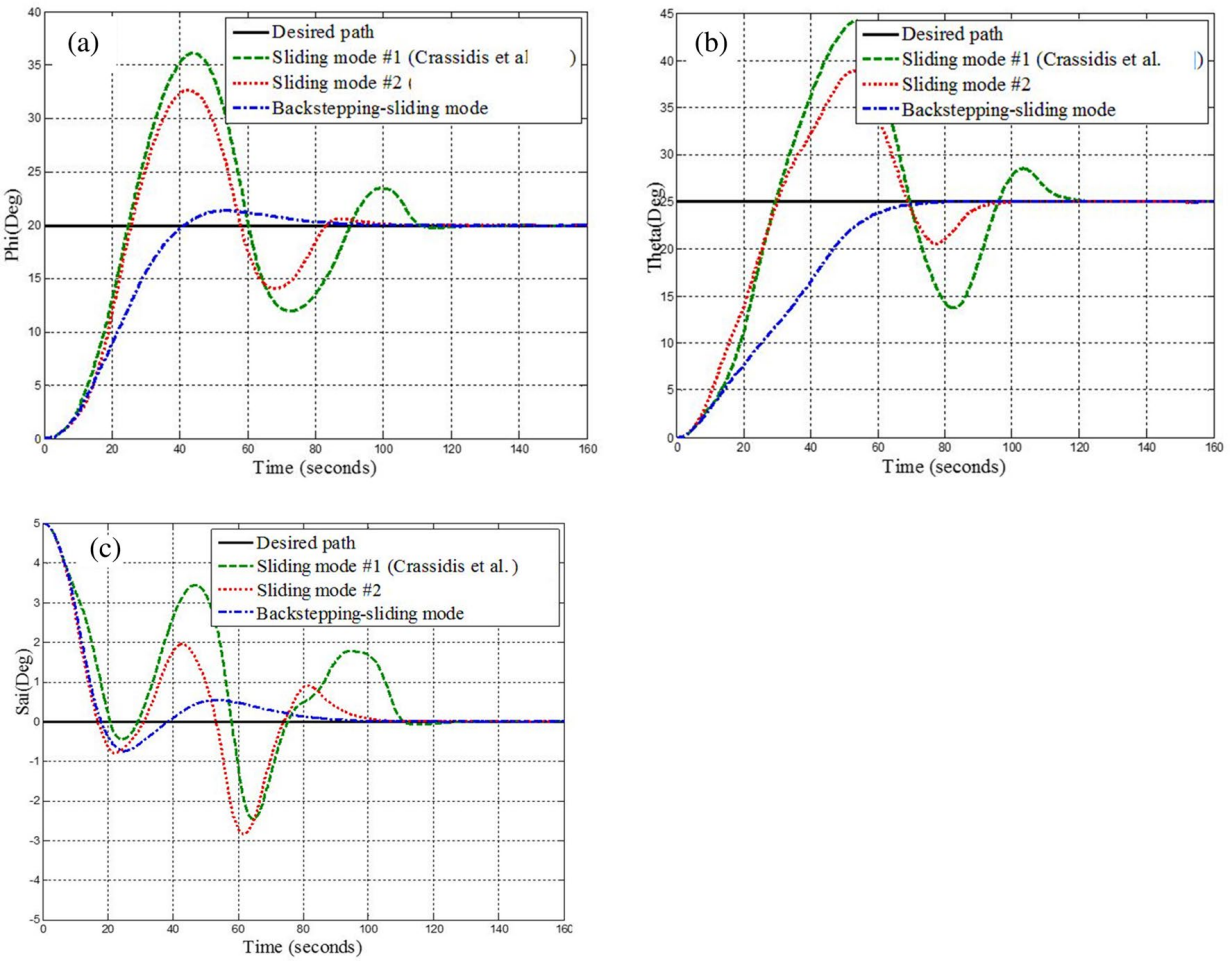
Simulation results for sliding mode and back-stepping sliding mode controller in the presence of reaction wheel actuator.

**Figure 9:**
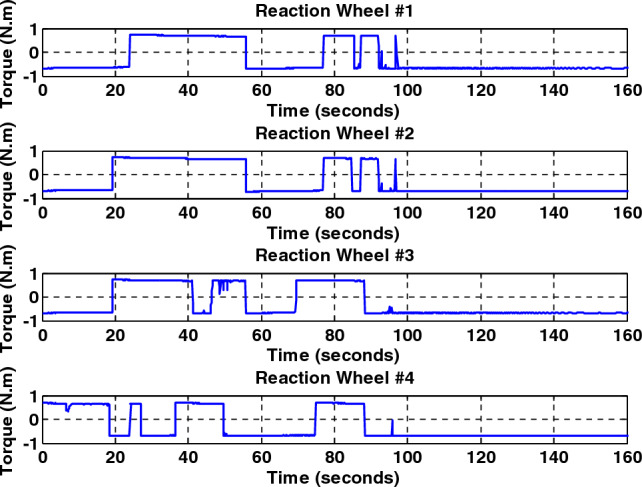
Torque generated by fault-free reaction wheels for back-stepping sliding mode controller.

**Figure 10:**
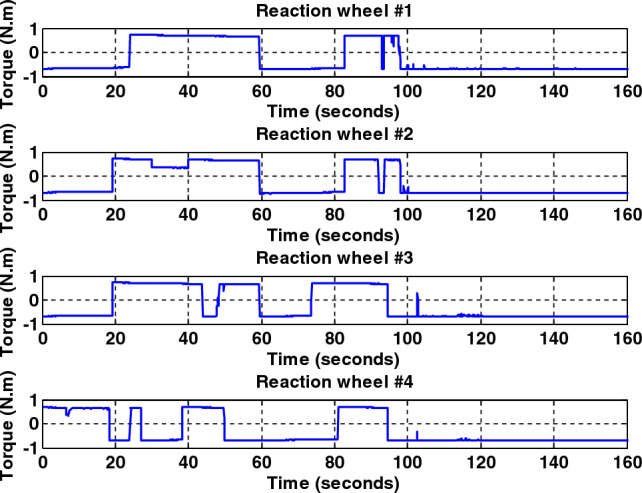
Torque generated by the wheels with the presence of a fault in the second wheel in thirty to forty seconds for the back-stepping sliding mode controller.

**Figure 11:**
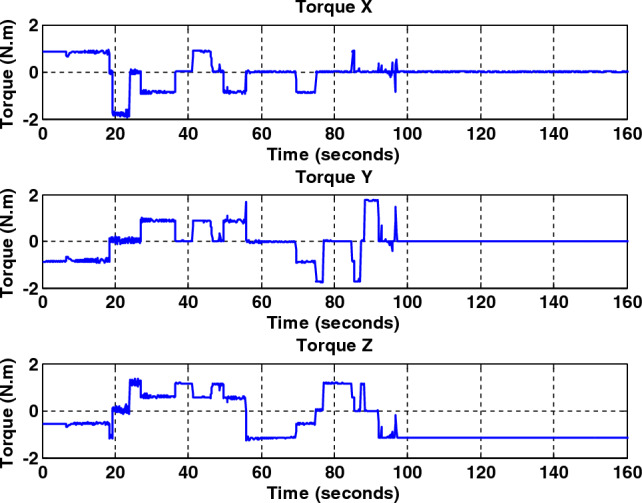
Total output torque for all axes without fault for back-stepping sliding mode controller.

**Figure 12:**
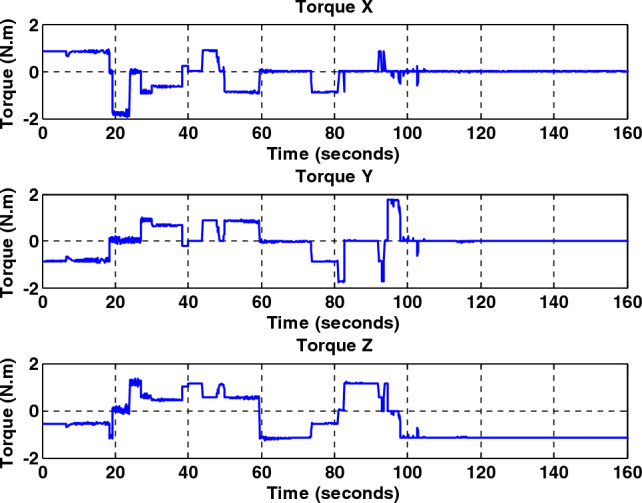
Total output torque for all axes with a faulty wheel #2 for back-stepping sliding mode controller.

Simulation results for the two controllers designed in the previous section and the sliding mode controller provided by Crassidis^27^ are presented in Fig. 8. The actuator used was the reaction wheel in a time of thirty to forty seconds, the reaction wheel number two is faulty and exerts fifty percent less torque than required by the spacecraft. As shown in Fig. 8, all three controllers stabilize the system despite the faults due to their robustness to disturbances and faults, but the back-stepping sliding mode control due to the dynamics of the actuator in its design, Has better performance and converges to the desired path with less and shorter error as well as faster. Also, since the maximum torque produced by the reaction wheels is limited, it takes approximately a long time (about one hundred seconds) for the spacecraft to reach the desired attitude. Therefore, it can be seen that all three controllers, using the reaction wheel alone, can provide accuracy (less than 0.03°) and fault tolerance, but the speed of convergence to the desired attitude is equal to one hundred seconds. It is not recommended to use this actuator when the maneuver time is short. Also, the output torque is given by the wheels and for different axes, with the presence of a fault and without the presence of a fault in Figs. 9, 10, 11, 12, for the back-stepping sliding mode control. It is observed that with the occurrence of a fault in wheel number two, the torque produced by the other wheels is changed so that the sum of the torque applied to each axis remains constant.

(B) Thrusters.

**Figure 13:**
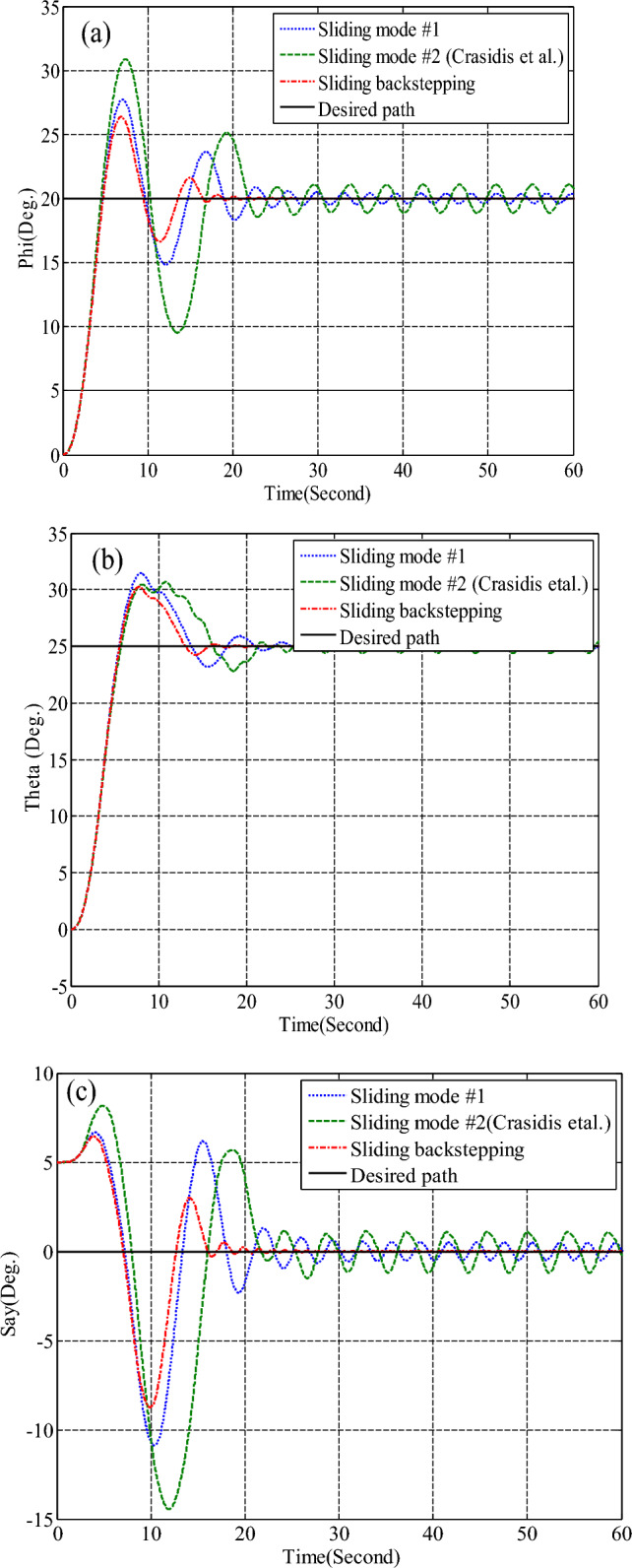
Simulation results for sliding mode and back-stepping sliding mode controller in the presence of a thruster actuator.

**Figure 14:**
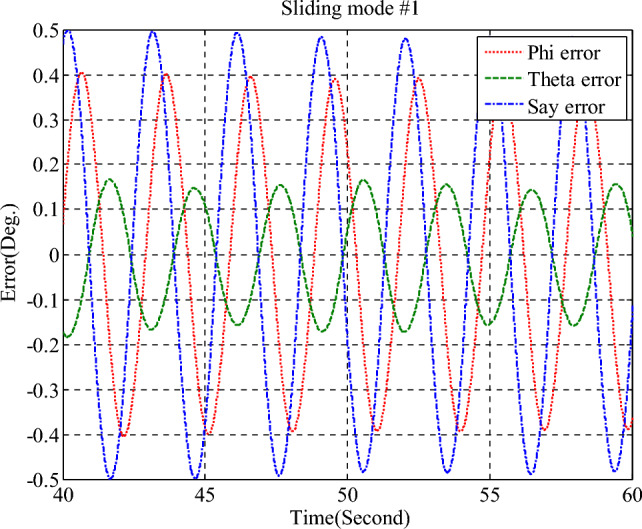
Steady-state error for sliding mode controller for all three axles in the presence of a thruster.

**Figure 15:**
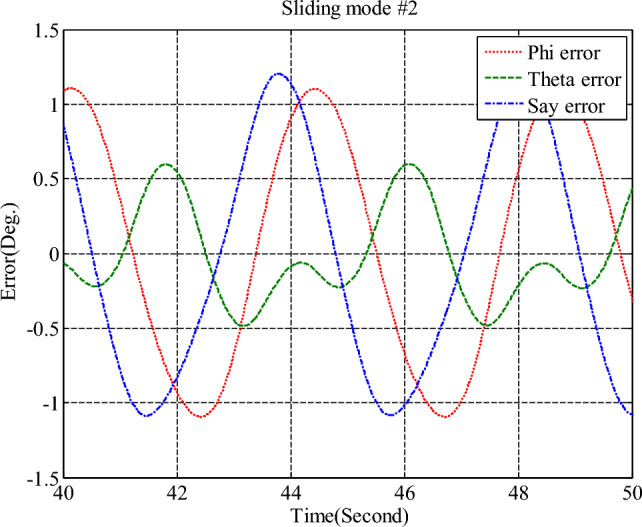
Steady-state error for sliding mode controller designed in reference^27^, for all axes in the presence of a thruster.

**Figure 16:**
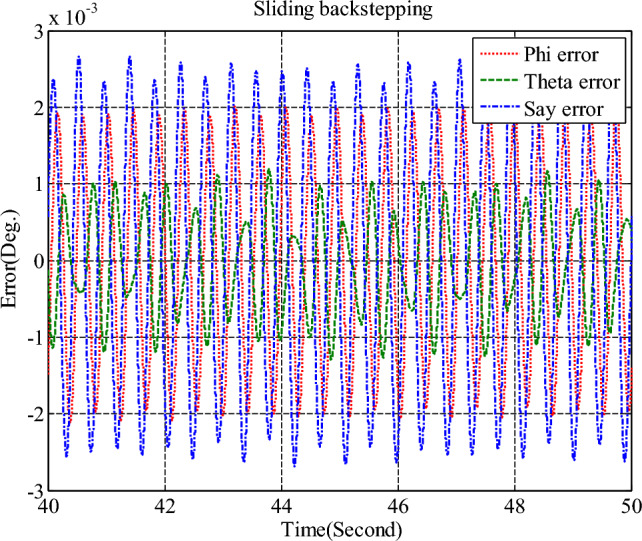
Steady-state error for back-stepping sliding mode controller, for all axes in the presence of a thruster.

**Figure 17:**
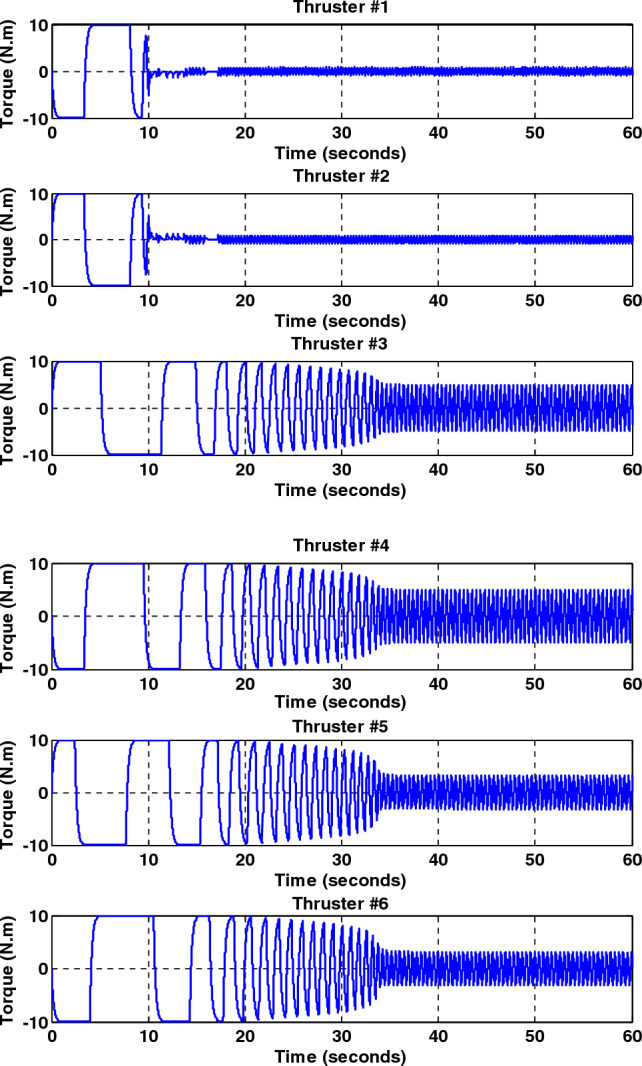
Torque generated by thrusters without fault for back-stepping sliding mode controller.

**Figure 18:**
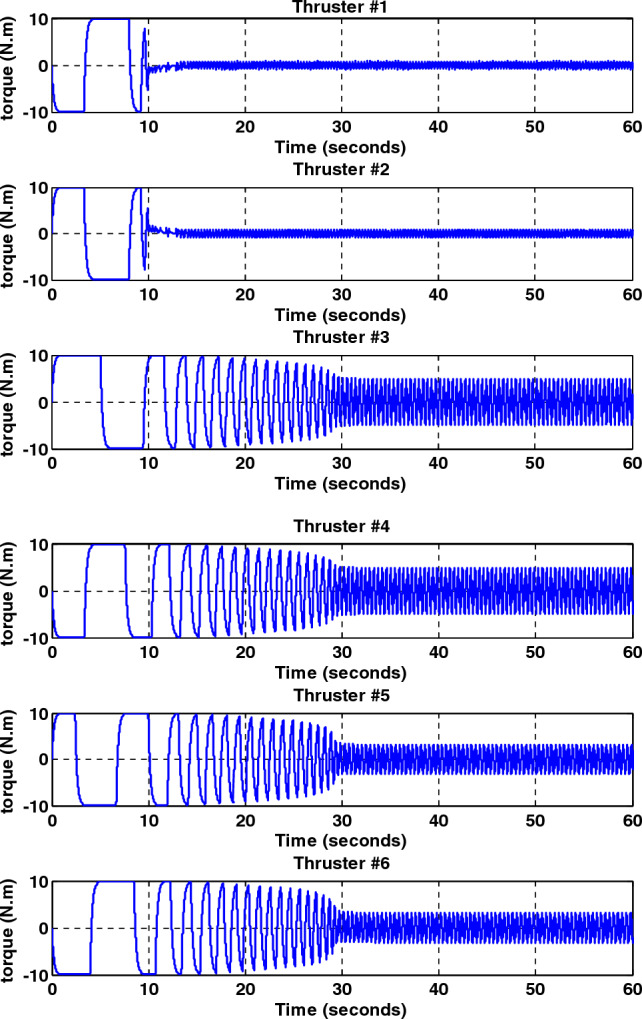
The torque produced by the thrusters with the presence of a fault in the X-axis thrusters for the back-stepping sliding mode controller.

**Figure 19:**
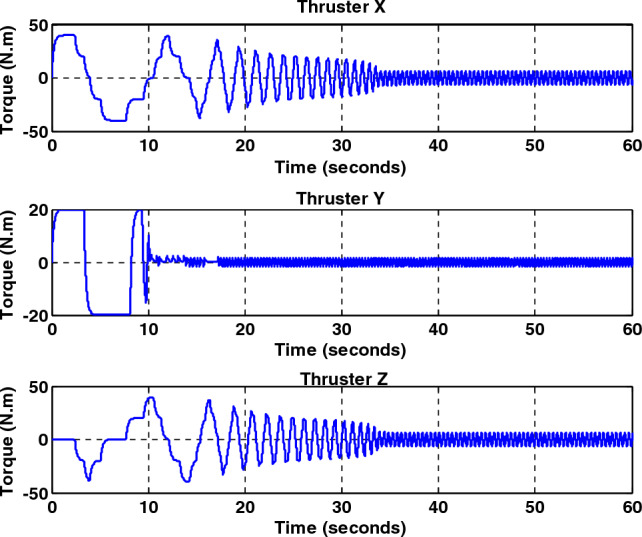
Total output torque for all axes without fault for back-stepping sliding mode controller.

**Figure 20:**
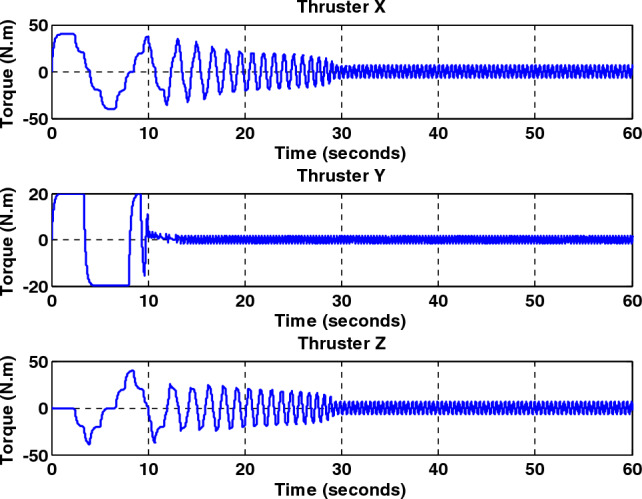
Total output torque for all axes with fault presence in X-axis thrusters for back-stepping sliding mode controller.

Simulation results for the sliding mode controller and the back-stepping sliding mode controller designed and also the sliding mode controller provided by^27^ for when the thruster actuator was employed are shown in Fig. 13. In four to fifteen seconds the x-axis thruster has a fault and applies one-half more torque to the spacecraft. As will be seen, when the actuator used was the reaction wheel, all three controllers stabilized the system despite the presence of a fault in the actuator, but the back-stepping sliding mode controller performed better with less error and converged to the specified state faster. Also, unlike when the reaction wheel was used, because the thruster is capable of manufacturing greater torque, the convergence time is shorter. But because of the heartbeat nature of the thruster, its steady state error isn’t zero, unlike the reaction wheel. The steady-state error for all three controllers is shown in Figs. 14, 15, 16. Therefore, all three controllers are fault-tolerant, but only the back-stepping sliding mode controller can meet the high control accuracy (0.03°) for sensitive maneuvers. Also, the torques produced by the thrusters are shown in Figs. 17, 18, 19, 20, for the back-stepping sliding mode. The results show that when the fault occurs, the controller changes the torque of each thruster, so the output of the torque applied to the axes is kept constant.

## Design of fault-tolerant attitude determination

In this section, to design a fault-tolerant attitude estimation system, the design of an Unscented Kalman filter is presented and how to use it in a Federated structure is described.

### Unscented Kalman filter

Instead of linearizing the equations to approximate the nonlinear model, EKF and UKF generate a limited number of points called sigma points. These points are transformed into a series of new points using nonlinear equations. The system states matrix and their dependent covariance error are numerically determined based on the mean values and covariance of the altered sigma points^41^

Suppose the model of a nonlinear system can be expressed as the following relation.

36 $$ \begin{array}{*{20}l} {X_{k + 1} = f(X_{k} ,t_{k} ) + W_{k} } \hfill & {W_{k} \approx N(0,Q_{k} )} \hfill \\ {Z_{k} = h(X_{k} ,t_{k} ) + V_{k} } \hfill & {V_{k} \approx N(0,R_{k} )} \hfill \\ \end{array} $$

In Eq. (36) $X_{k}$, is states vector of the system at the time $t_{k}$ and $Z_{k} ,\;W_{k} ,\;V_{k}$ represents the measurement vector, state noise process vector, and measurement noise vector, respectively. *h* and *f* are nonlinear process models. The UKF process is similar to the standard Kalman filter process, to which a predictive return loop has been added.

### Unscented Kalman filter design for spacecraft

As described in the previous section, state vectors, and nonlinear models must be specified for estimating states by local filters of the Unscented type. The state vectors are selected based on the variables to be estimated. Our goal is to estimate the attitude of the spacecraft and its angular velocities used in the controller. Therefore, the state vector is defined as follows, which includes the angular velocities of the spacecraft and the Euler angles.

37 $$ x = \left\lfloor {\begin{array}{*{20}l} {\omega_{x} } \hfill & {\omega_{y} } \hfill & {\omega_{z} } \hfill & \psi \hfill & \theta \hfill & \phi \hfill \\ \end{array} } \right\rfloor $$

Also, according to the dynamic and kinematic equations in the state space, the nonlinear function $f$ can be determined as follows:

38 $$ \dot{\omega }_{x} = \dot{x}_{1} = (I_{y} - I_{z} )I_{x}^{ - 1} x_{2} x_{3} + M_{x} I_{x}^{ - 1} $$

39 $$ \dot{\omega }_{y} = \dot{x}_{2} = (I_{z} - I_{x} )I_{y}^{ - 1} x_{1} x_{3} + M_{y} I_{y}^{ - 1} $$

40 $$ \dot{\omega }_{z} = \dot{x}_{3} = (I_{x} - I_{y} )I_{z}^{ - 1} x_{1} x_{2} + M_{z} I_{z}^{ - 1} $$

41 $$ \dot{\psi } = \dot{x}_{4} = \frac{{\sin (x_{6} )}}{{\cos (x_{5} )}}x_{2} + \frac{{\cos (x_{6} )}}{{\cos (x_{5} )}}x_{3} $$

42 $$ \dot{\theta } = \dot{x}_{5} = \cos (x_{6} )x_{2} - \sin (x_{6} )x_{3} $$

43 $$ \dot{\phi } = \dot{x}_{6} = x_{1} + \sin (x_{6} )\tan (x_{5} )x_{2} - \cos (x_{6} )\tan (x_{5} )x_{3} $$

The function $h$, shows a nonlinear relationship between the outputs measured by the sensors and the system states. Since our sensors also measure system states directly, $h$ is obtained for the gyro and star tracker according to Eqs. (44) and 45, respectively

44 $$ h_{gyro} = \left[ {\begin{array}{*{20}c} 1 & 0 & 0 & 0 & 0 & 0 \\ 0 & 1 & 0 & 0 & 0 & 0 \\ 0 & 0 & 1 & 0 & 0 & 0 \\ \end{array} } \right]\left[ {\begin{array}{*{20}c} {\omega_{x} } & {\omega_{y} } & {\omega_{z} } & \psi & \theta & \phi \\ \end{array} } \right]^{T} $$

45 $$h_{star} = \left[ {\begin{array}{*{20}c} 0 & 0 & 0 & 1 & 0 & 0 \\ 0 & 0 & 0 & 0 & 1 & 0 \\ 0 & 0 & 0 & 0 & 0 & 1 \\ \end{array} } \right]\left[ {\begin{array}{*{20}c} {\omega_{x} } & {\omega_{y} } & {\omega_{z} } & \psi & \theta & \phi \\ \end{array} } \right]^{T}$$

### Federated Kalman filter structure

**Figure 21:**
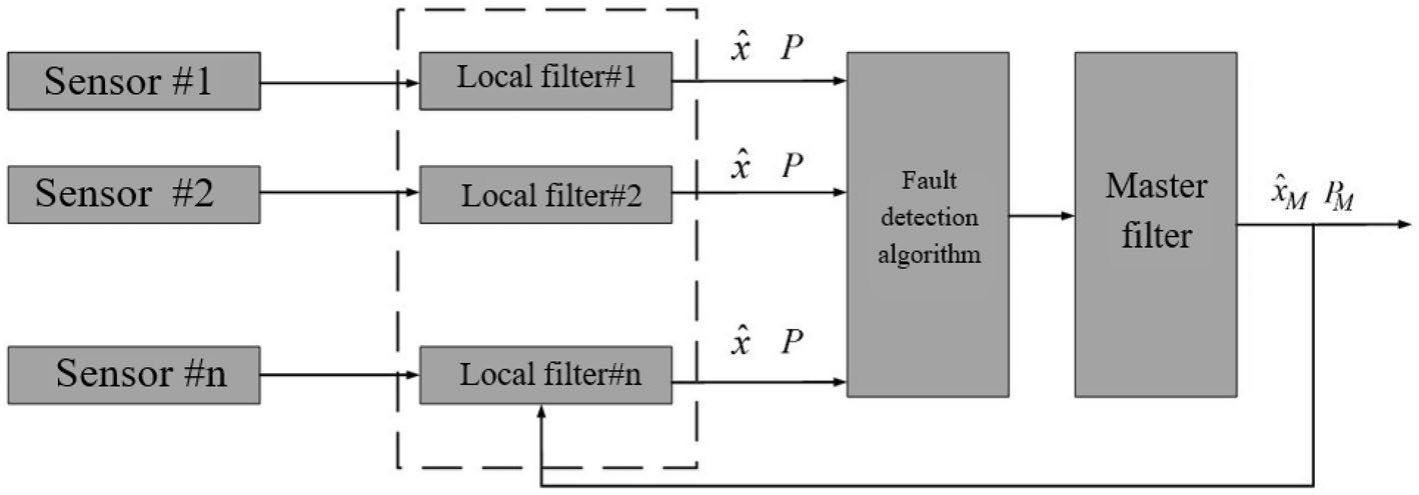
Federated filter structure.

Federated filters consist of two parts: local filters and the main filter. Local filters are applied in parallel and independently of each other, and the results estimated by them are combined in the main filter. In each local filter, an estimate of system states is made using local sensor measurements. The main filter uses local filter estimates to update the overall state estimate in the data integration process and uses the results to initialize local filters (Fig. 21). This design has several features, such as the ability to detect and isolate faults in any local sensor, and also that the main filter is not affected by the failure of the local sensor^41,42^ in other words, it is resistant to faults. Once the local filters have estimated the system states, these states are checked by a fault detection algorithm described in the next section, and if there is a fault, the data is removed and not used in the main filter.

The main filter works at the same speed as local filters. If all local estimates are separate, the overall estimate is obtained by the main filter from the following equation.

46 $$\hat{x}_{M} = P_{M} \left\{ {P_{1}^{ - 1} \hat{x}_{1} + P_{2}^{ - 1} \hat{x}_{2} + \cdots + P_{N}^{ - 1} \hat{x}_{N} } \right\}$$

47 $$P_{M}^{ - 1} = P_{1}^{ - 1} + P_{2}^{ - 1} + \cdots + P_{N}^{ - 1}$$

$\hat{x}_{i}$, ${P}_{i}$ are an estimation and covariance matrix of i-th local filter and $P_{M}^{ - 1}$ is an Information matrix. Note that the overall estimate is the sum of the local estimates with their linear weight composition.

The UKF filter in an organized structure can generate accurate and robust state estimation values without disrupting the mission by using a fault detection algorithm. Fault detection usually requires the continuous display of measured output data. Normally, the output data follows a known evolutionary pattern with random and finite perturbations and measured noise. However, when a fault occurs in the sensor, for the measured output data, the nominal value of the data evolution pattern changes. Fault detection algorithms are typically based on examining the discrepancy between evolutionary patterns and the amount of data measured^43^. The value of the sensitivity factor used is obtained from the following equation.

48 $$S_{i} = \left( {\hat{x}_{i} - \hat{x}_{M} } \right)^{T} \left( {P_{i} + P_{M} } \right)\left( {\hat{x}_{i} - \hat{x}_{M} } \right)$$

When $S_{i}$ it is less than the threshold value, the i-th sensor is considered a healthy sensor, and the output data can be used to estimate the final state. However, if the value is greater than the threshold value, it indicates that the i-th sensor has a fault and the output data is not being used. The threshold value is selected based on the distribution of Chi-squares and is optimized based on different applications in the experiments.

## Simulation results

In this section, to evaluate the performance of the fault-tolerant attitude estimation system, simulations are performed for three modes: no fault, faulty sensor but no fault detection and isolation algorithm, and faulty sensor with the presence of fault detection and isolation algorithm. The attitude determination system includes two star trackers and a gyro sensor, and the attitude determination and control system has the task of bringing the spacecraft from the initial angle of zero degrees to the desired angles that are assumed to be equal to $\psi_{d} = 20^{ \circ } ,\theta_{d} = 25^{ \circ } ,\phi_{d} = 0^{ \circ }$ . The system parameters are shown in Table 2.

**Table 2:** System parameters.

Quantity	Parameter	
$\left[ {1000 , 500 , 700} \right] kg\;m^{2}$	$\left[ {J_{x} ,J_{y} ,J_{z} } \right]$	Dynamics of satellite
$\left[ {0,0,5} \right] deg$	$\left[ {\phi_{0} ,\theta_{0} ,\psi_{0} } \right]$initial attitude	
$\left[ {20,25,0} \right] deg$	$\left[ {\phi ,\theta ,\psi } \right]$desired attitude	
$\left( {\frac{0.03}{3}} \right)^{2} deg$	Noise measurement	Star tracker
$3 \times 10^{ - 6} \left( {\frac{rad}{{sec^{0.5} }}} \right)$	Sensor noise	gyro

(A) A.The results of the system without the fault estimation algorithm can be seen in the Figs. 21, 22 and 23, the system works properly without the presence of a fault and follows the desired path. These figures also show that the sensors and filters provide an accurate local estimate of the spacecraft, and since there is no fault in the sensor, all data is used in the main filter.

**Figure 22:**
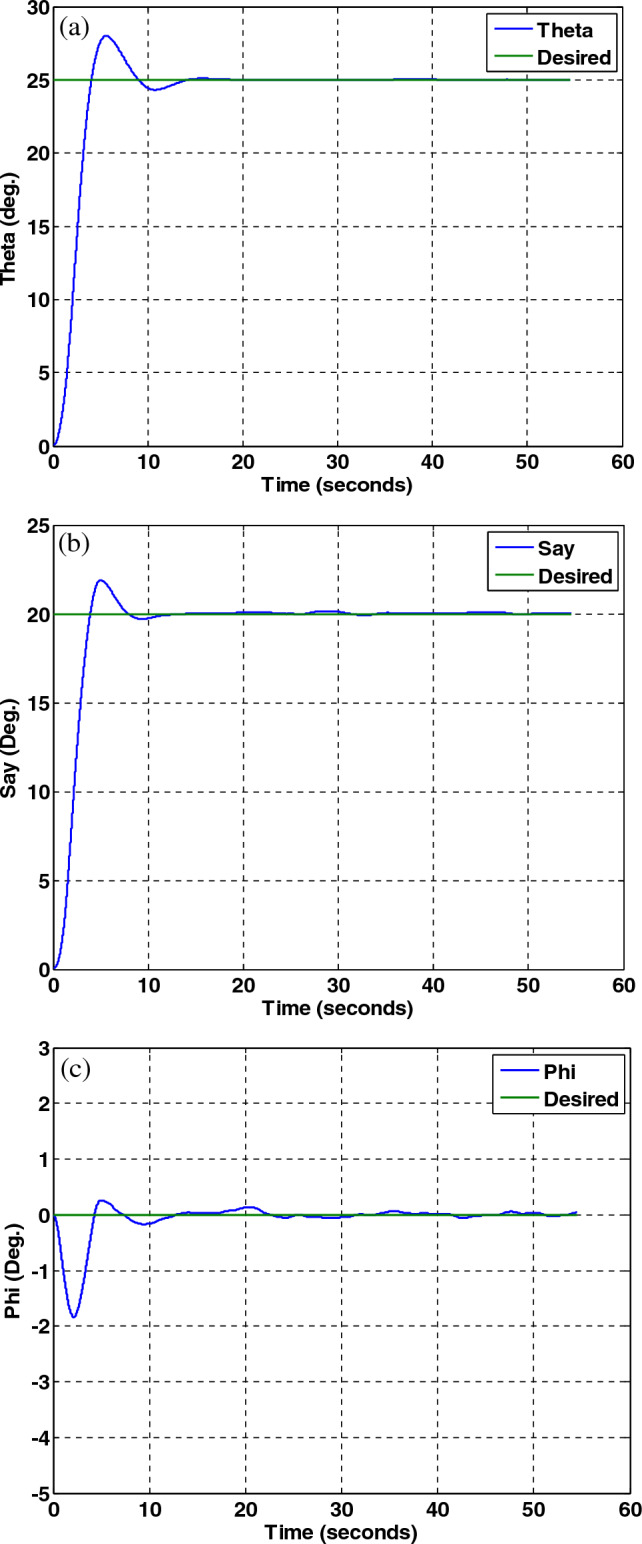
Performance of attitude determination system for all axes without fault in the sensor and without fault determination algorithm.

**Figure 23:**
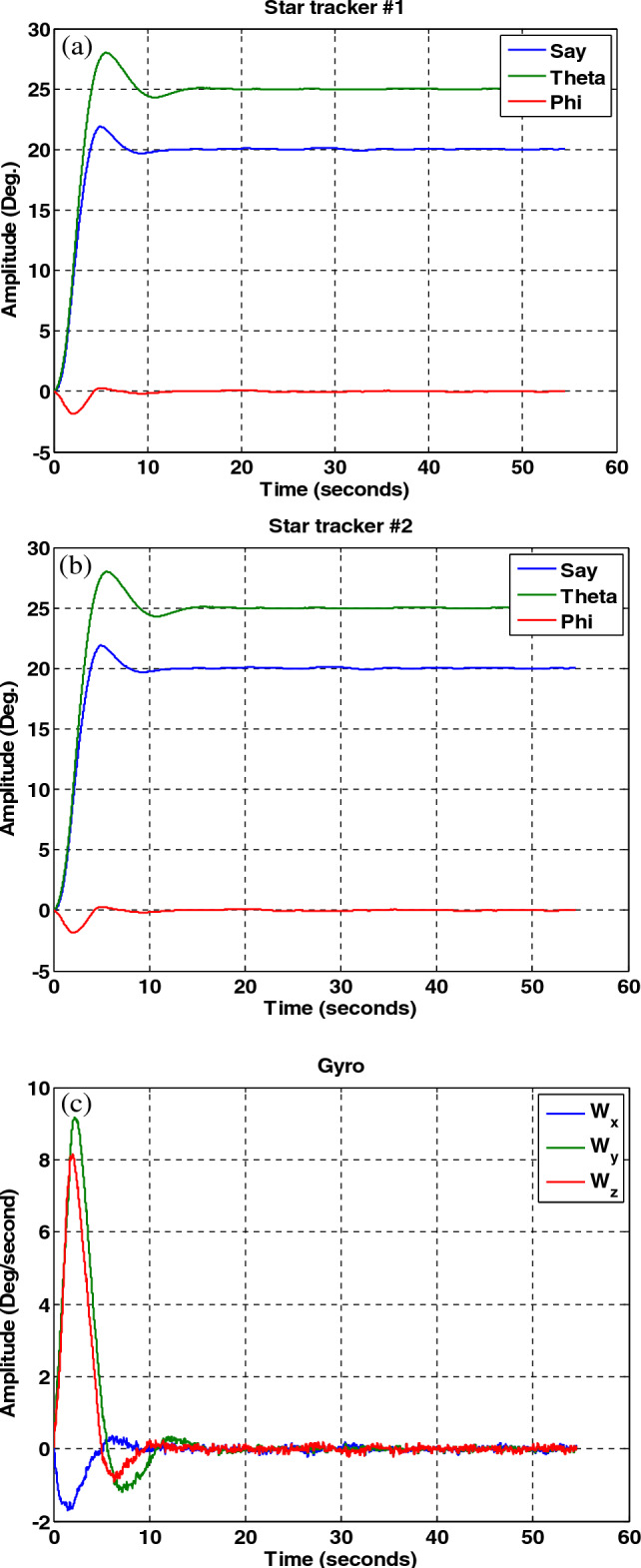
Sensor output, no fault in the sensors and no fault detection algorithm.
(B) The results of the attitude estimation system in the presence of a fault in the sensor without a fault determination algorithm.

**Figure 24:**
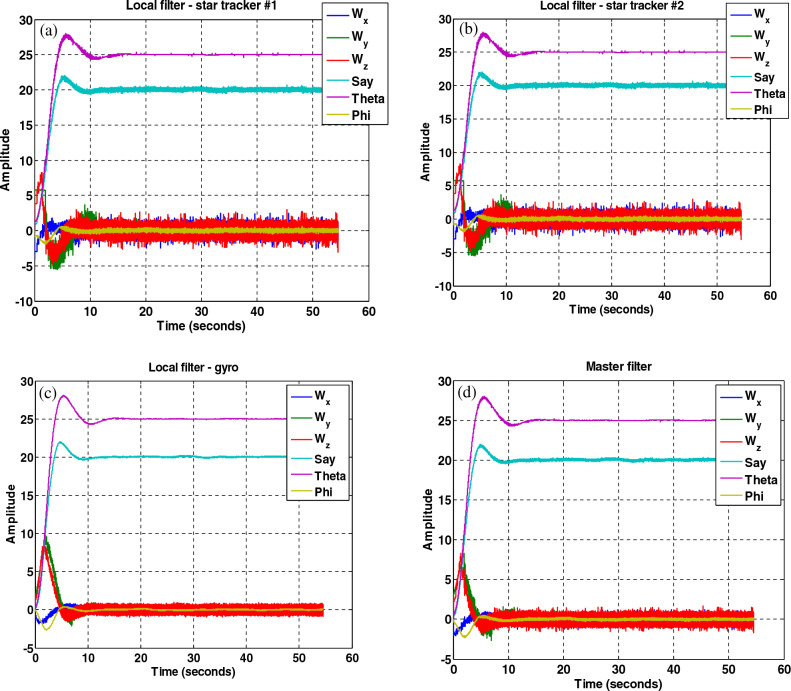
Estimation of states by local and main filters without fault in sensors and without fault determination algorithm.

**Figure 25:**
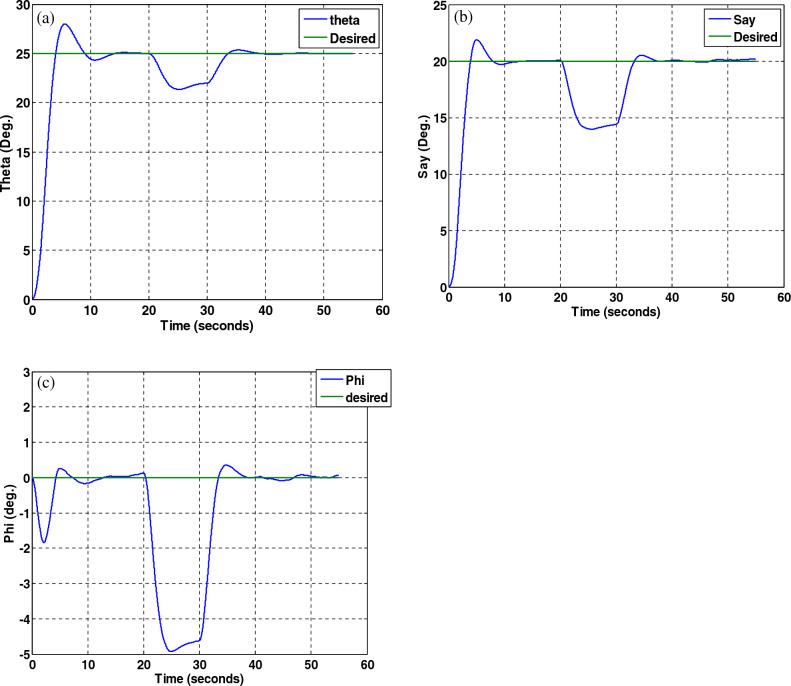
System performance in tracking for all axes with the presence of a fault in the number one star tracker without a fault determination algorithm.

**Figure 26:**
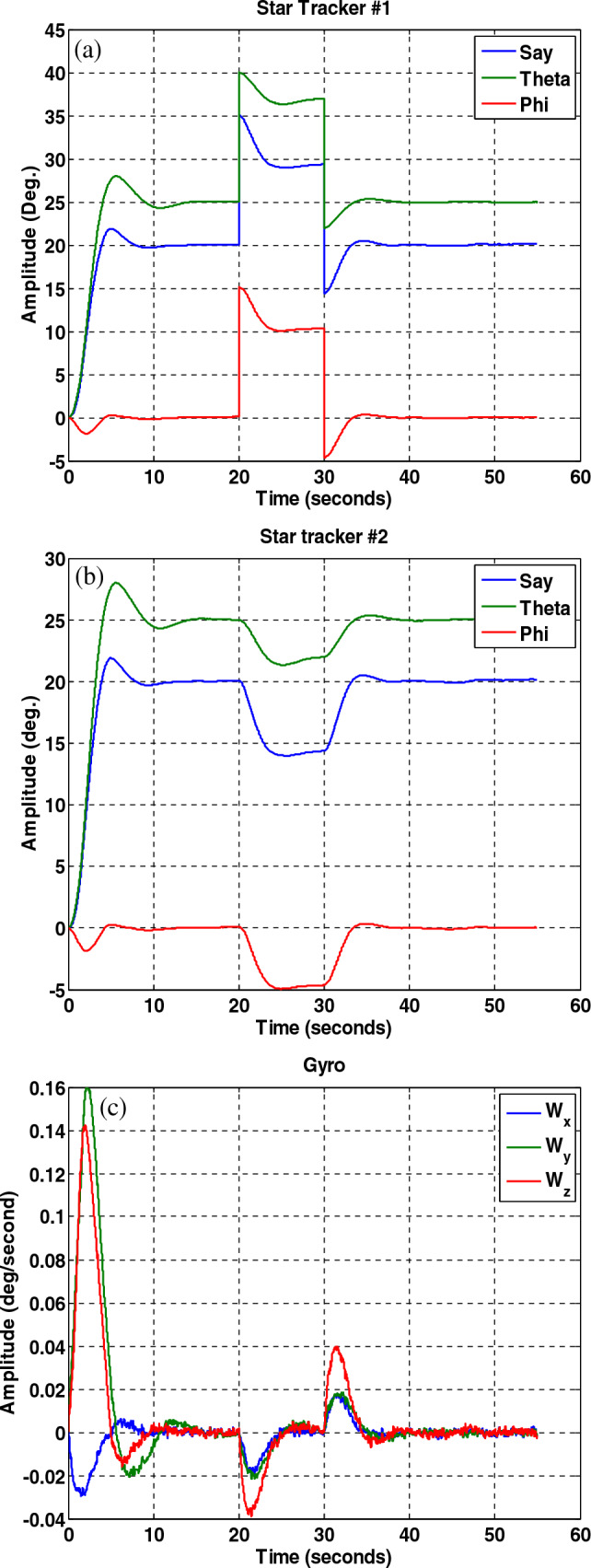
Outputs of sensors with the presence of a fault in the number one, star tracker and without a fault determination algorithm.

In this section, the results are shown for a 15-degree fault in the number one star tracker. Note that since the presence of a fault in other sensors leads to similar results, the same results have been avoided for them, and only the fault in the number one sensor Has been considered. The results in Figs. 24, 25, 26 show that although the local filters of Star Tracker No. 2 and the gyro correctly estimate the state of the system, the entire system will not be able to perform its mission properly at that time, because of a faulty sensor.

(C) The results of the attitude estimation system in the presence of a fault in the sensor with a fault determination algorithm.

**Figure 27:**
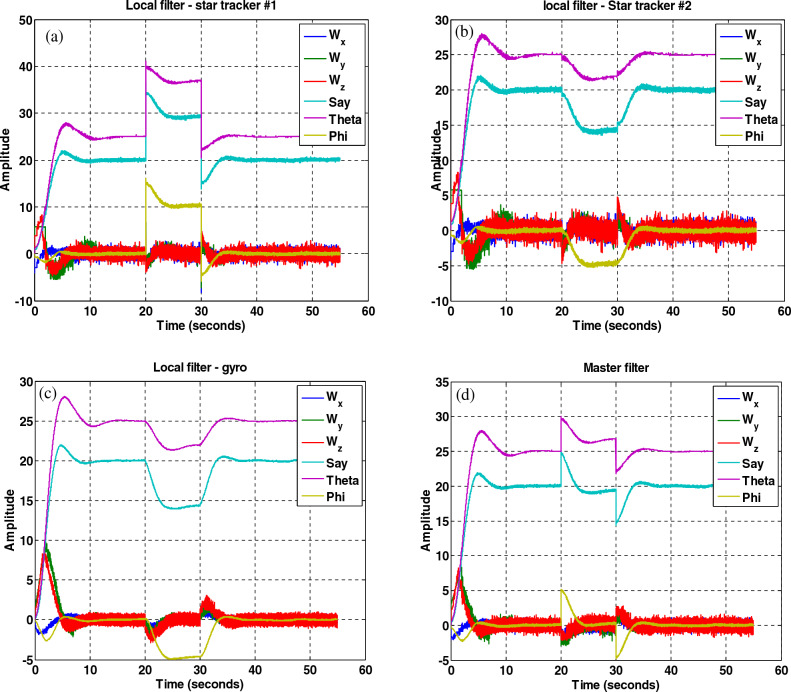
States estimation by local filters and master filter with the presence of a fault in the number one star tracker and without a fault determination algorithm.

**Figure 28:**
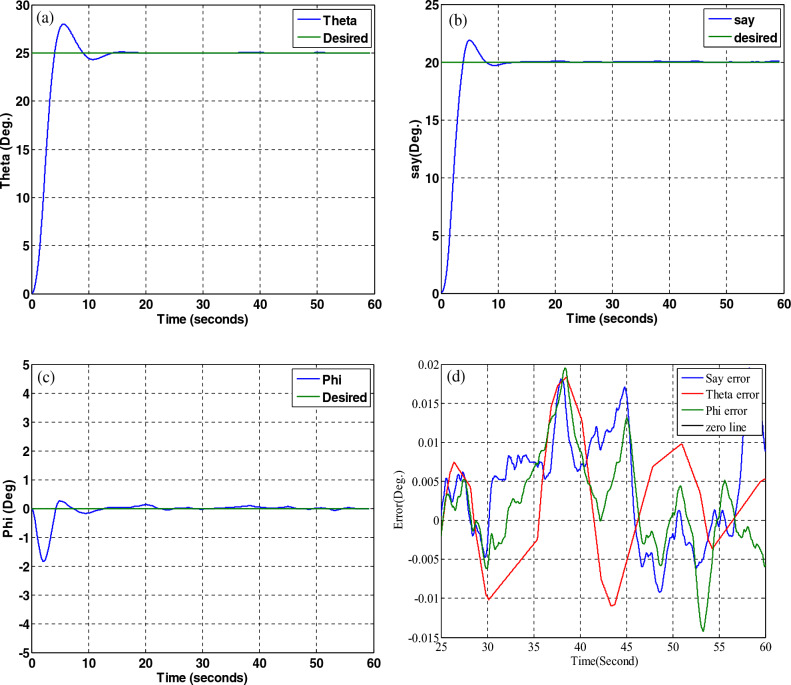
System performance in tracking for all axes with the presence of a fault in the number one star tracker with a fault determination algorithm.

**Figure 29:**
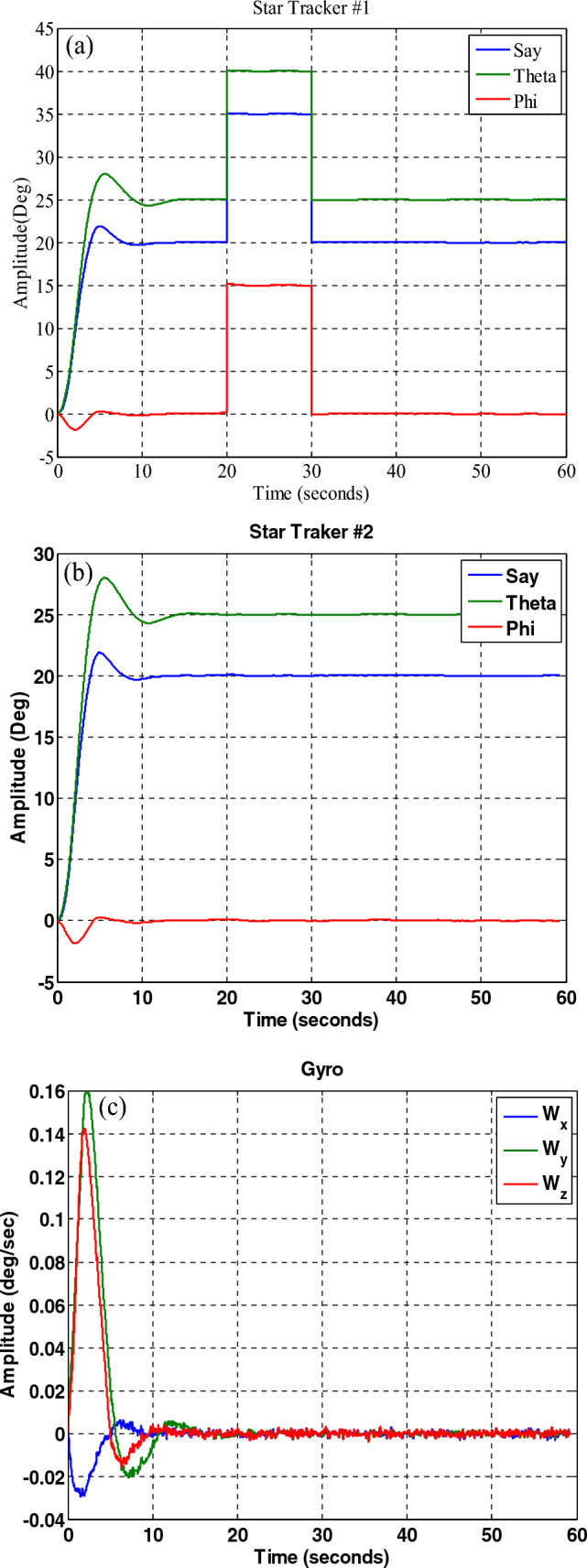
Outputs of sensors with the presence of a fault in the number one star tracker with a fault determination algorithm.

**Figure 30:**
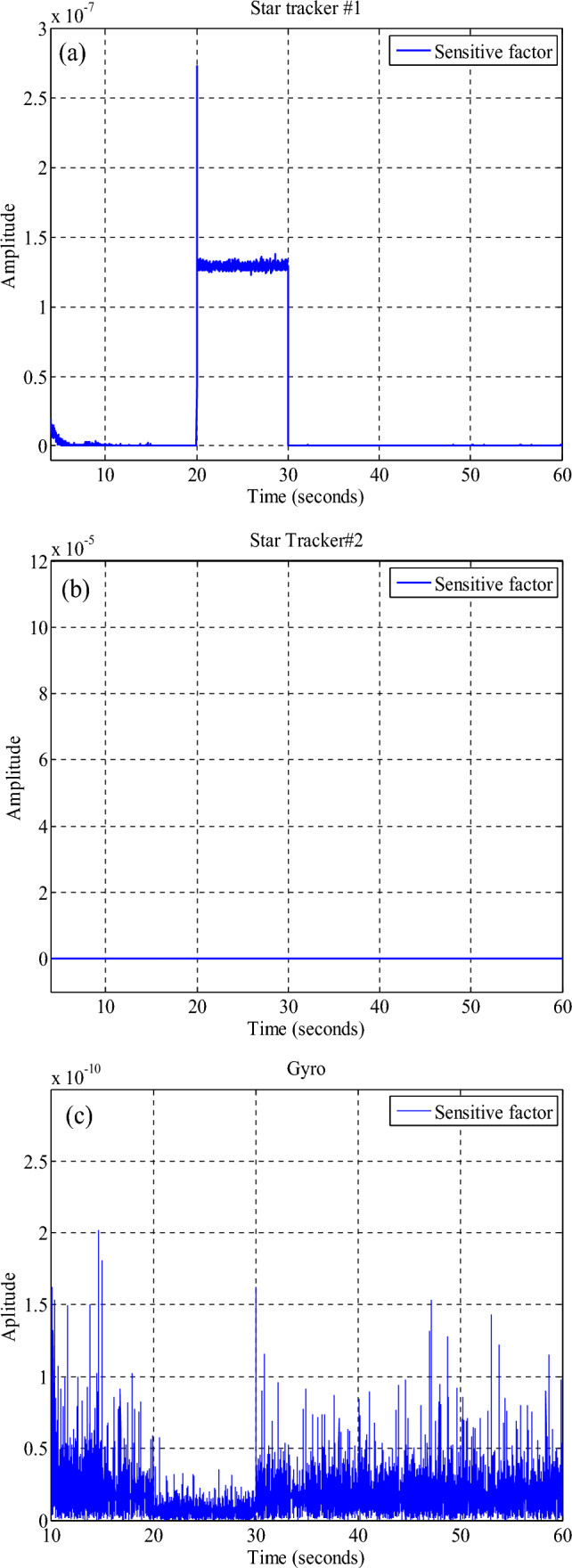
Sensitive factor for all sensors with the presence of a fault in the number one star tracker.

**Figure 31:**
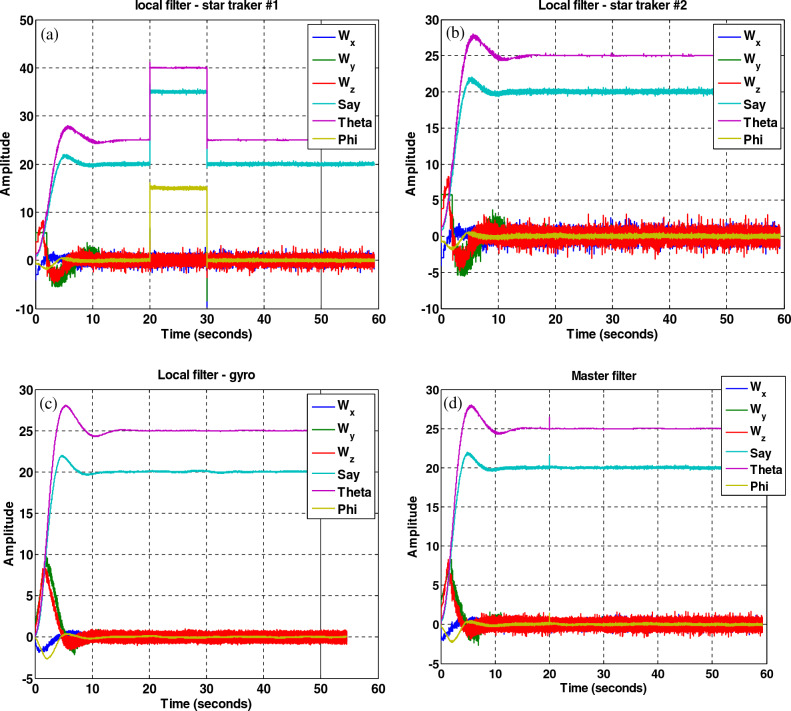
States estimation by local filters and master filter with the presence of a fault in the number one star tracker with a fault determination algorithm.

The results in Figs. 27, 28, 29, 30, 31 has been shown. The results show that although there is a fault in the star tracker number one sensor for a period of 20–30 s because the attitude determination system detects this fault and removes the number one sensor from the attitude estimation algorithm, the system correctly performs its mission and the desired attitude has been tracked.

**Figure 32:**
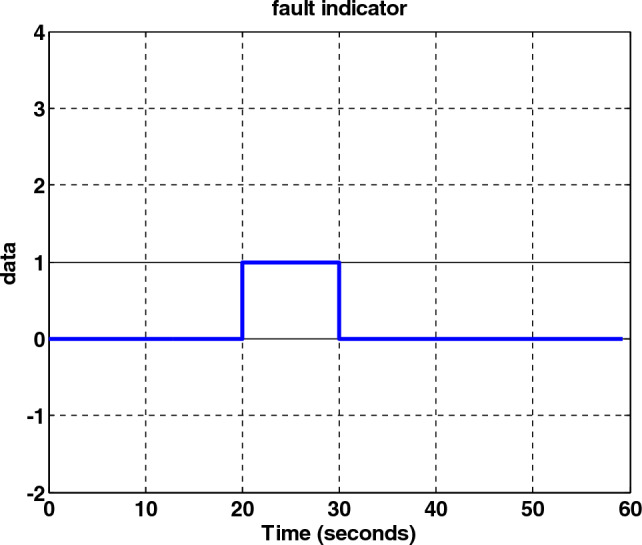
Fault indicator.

Table 3 is intended to isolate the fault. As can be seen from the results, the system has quickly identified the faulty sensor. The indicator of the faulty sensor is shown in Fig. 32.

**Table 3:** Display logic to indicate a fault.

Indicator	Type of fault
0	No fault
1	Star Tracker #1
2	Star Tracker #2
3	Gyro

As it turned out from the results, the fault-tolerant attitude estimation system has two important features, high speed in determining and eliminating the faulty sensor, as well as high accuracy. Therefore, it is suitable for use in the attitude determination system of a satellite.

## Simultaneous fault in actuator and sensor

In this section, to evaluate the performance of fault tolerant control systems and fault tolerant attitude estimation, simulation is performed when the fault occurs simultaneously in the sensor and the actuator. For this purpose, in 20–30 s, for the number one star tracker, a 15-degree increase in showing the attitude and for the x-axis thruster, a fifty percent increase in the required torque production is considered as a fault.

**Figure 33:**
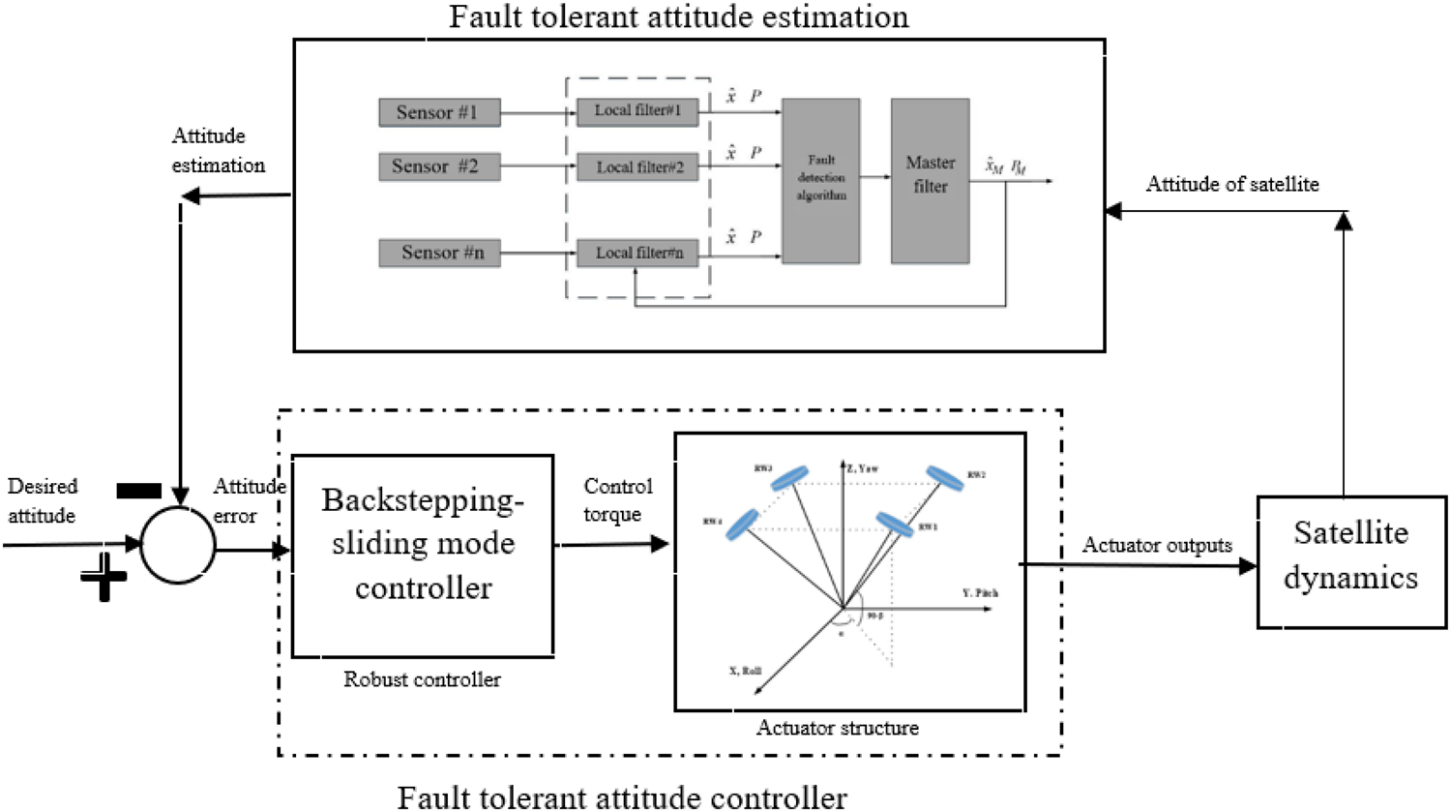
Fault-tolerant controller and estimation.

The structure of the attitude controller and estimation are shown in Fig. 33.

**Figure 34:**
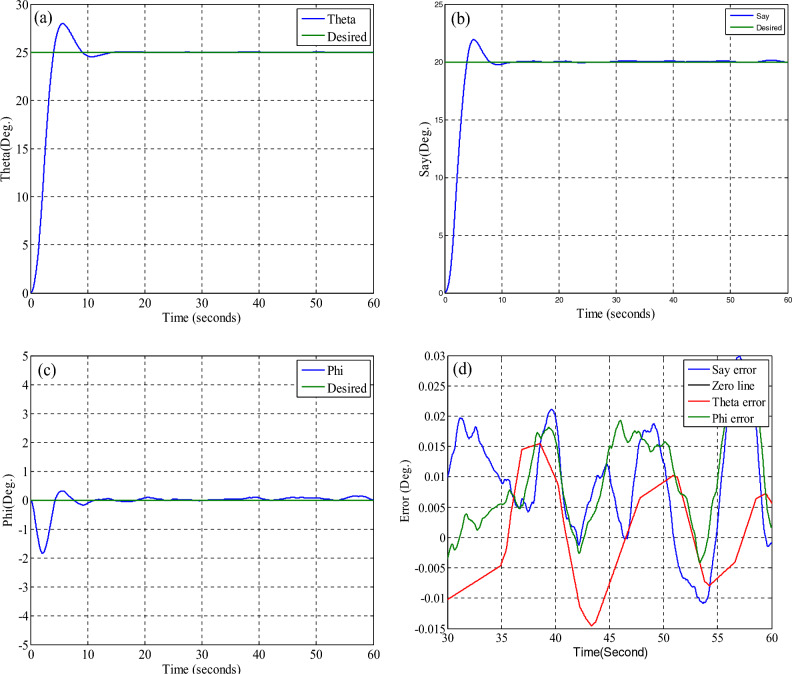
System performance in tracking for all axes with the presence of a fault in the number one star tracker and X-axis thrusters.

The simulation results are given in Fig. 34. As can be seen from the results, despite considering the fault simultaneously for the sensor and the actuator, the fault tolerant control systems and fault tolerant attitude estimation work properly together.

## Conclusions

A robust attitude control algorithm is developed based on the back‌-stepping sliding mode control for a satellite by Considering the dynamics of the actuators and the asymptotic stability of the proposed algorithm has been proved based on Lyapunov theory. Then a fault-tolerant attitude estimation system is designed based on federated unscented Kalman filters that can be effectively employed to detect and isolate sensor faults. According to the dynamics of the system, the simulation results show that despite the fault in the actuators and sensors at the same time, the satellite can maintain its desired attitude and stability, while if any of these systems did not exist, the satellite’s mission would be lost.

## Data Availability

All data generated or analyzed during this study are included in this published article but if you need more data, please contact the corresponding author.
